# Supplementary material for: Effector loading onto the VgrG carrier activates type VI secretion system assembly
Source: EMBO Rep. 2019 Dec 5;21(1):e47961. doi: 10.15252/embr.201947961 (PMC6945064; doi:10.15252/embr.201947961)
Supplement: Supplementary file 1 — Appendix [file EMBR-21-e47961-s001.pdf]

Table of Content

Appendix Table S1. Strains and plasmids used in this study----------p1

Appendix Table S2. Primers used in this study--------------------------p8

Cited references---p13

Appendix Table S1. Strains and plasmids used in this study

Strain	Lab strain name	Relevant characteristics and/or purpose	Reference/ Source
A. tumefaciens			
C58	EML530	Wild type virulent nopaline type strain, isolated from a cherry gall	Eugene Nester
Δ*tssL*	EML1073	*tssL* deletion mutant	[3]
Δ*tde1-tdi1*	EML3392	deletion mutant of *tde1-tdi1* effector-immunity gene pair	[4]
Δ*tde2-tdi2*	EML3551	deletion mutant of *tde2-tdi2* effector-immunity gene pair	[4]
Δ*tae-tai*	EML3553	deletion mutant of *tae-tai* effector-immunity gene pair	[5]
Δ*tdei*	EML3559	Double deletion mutant of *tde1-tdi1* and *tde2-tdi2* effector-immunity gene pairs	[4]
Δ*tde1-tdi1*Δ*tae-tai*	EML3555	Double deletion mutant of *tde1-tdi1* and *tae-tai* effector-immunity gene pairs	This study
Δ*tde2-tdi2*Δ*tae-tai*	EML3557	Double deletion mutant of *tae-tai* and *tde2-tdi2* effector-immunity gene pairs	This study
Δ3TIs	EML3561	Triple deletion mutant of *tae-tai*, *tde1-tdi1* and *tde2-tdi2* effector-immunity gene pairs	[4]
Δ*tap-1*	EML4290	Deletion mutant of Tde1 adaptor/chaperone gene *tap-1*	[4]
Δ*atu3641*	EML3406	Deletion mutant of Tde2 adaptor chaperone gene *atu3641*	[6]

Δ*G1*Δ*G2op*	EML5132	mutant with deletion of *vgrG1* gene and *vgrG2* operon	This study
Δ*G2op*	EML3677	mutant with deletion of *vgrG2* operon	This study
Δ*tdei*Δ*G1*Δ*G2op*	EML5130	mutant with deletion of *tde1-tdi1* effector-immunity gene pair, *vgrG1* gene, and *vgrG2* operon	This study
Δ*tssB*	EML1109	*tssB* deletion mutant	[5]
Δ*tssL*Δ*tssB*	EML5156	Double deletion mutant of *tssL* and *tssB*	This study
Δ*tdei*Δ*tssB*	EML5159	mutant with deletion of *tde1-tdi1* and *tde2-tdi2* effector-immunity gene pairs and *tssB* gene	This study
12D1	EML490	Wild type virulent strain	Clarence I Kado
12D1Δ*tssL*	EML4635	12D1 *tssL* deletion mutant	[9]
12D1Δ*v1*	EML4688	12D1 adapter deletion mutant	This study
12D1Δ*v3-4*	EML4679	12D1 putative effector-immunity pair deletion mutant	This study
1D1108	EML302	Wild type virulent nopaline type strain isolated from a *Euonymus* gall	Clarence I Kado
1D1108Δ*tssL*	EML4631	1D1108 *tssL* deletion mutant	[9]
1D1108Δ*v1-9*	EML4645	1D1108 with deletion of all predicted *vgrG*-associated genes	This study
1D1108Δ*v4*	EML4690	1D1108 adaptor/chaperone gene deletion mutant	This study
1D1108Δ*v6*	EML4692	1D1108 PAAR-like domain-encoding gene deletion mutant	This study

15955	EML459	Wild type virulent agropine type strain isolated from a tomato gall	Clarence I Kado
15955Δ*tssL*	EML4629	15955 *tssL* deletion mutant	[9]
15955Δ*v1-7*	EML4650	15955 with deletion of all predicted *vgrG*-associated genes	This study
Δ*tap-1*Δ*atu3641*		Double deletion mutant of *tap-1* and *atu3641*	This study
12D1Δ*tae-tai*		12D1 deletion mutant of *tae-tai* effector-immunity gene pair	This study
12D1Δ*v3-4*Δ*tae-tai*		12D1 double deletion mutant of *tae-tai* and *v3-v4* effector-immunity gene pairs	This study
Δ*tae-tai**		New deletion mutant of *tae-tai* effector-immunity gene pair	This study
Δ*tap-1*Δ*atu3641*Δ*tae-tai**		Triple deletion mutant of *tap-1*, *atu3641* and *tae-tai* effector-immunity gene pair	This study
Δ3TIs*		New triple deletion mutant of *tae-tai*, *tde1-tdi1* and *tde2-tdi2* effector-immunity gene pairs	This study
E. coli			
DH10B	EML455	Host for molecular cloning and target cell for bacterial competition assay	Invitrogen
Plasmids			
pRL662	EML315	Broad host range vector derived from pBBR1MCS-2, Gm^R	[7]
pTrc200	EML904	pVS1 origin, *$lacI^q$, trc* promoter, Sp^R	[8]

pTdei1	EML4277	*tde1* and *tdi1* in pTrc200, Sp^R	This study
pTde2*	EML4797	*tde2* catalytic site mutant in pRL662, Gm^R	This study
pTap-1	EML4255	*tap-1* in pTrc200, Sp^R	[4]
p3641	EML4785	atu3641 in pTrc200, Sp^R	[6]
pTrc-TssB-GFP	EML5154	*tssB* fused at its C-terminal coding portion to *gfp* in pTrc200, Sp^R	This study
pTrc-TssB	EML4043	*tssB* in pTrc200, Sp^R	This study
pBBR1-GFP	EML3	Broad-host range vector	[1]
pTae	EML1616	*tae* in pRL662, Gm^R	[4]
pTai	EML4228	*tai* in pTrc200, Sp^R	[5]
$pG1^{812}$	EML4567	*vgrG1* encoding VgrG1 variants with amino acid residue 813 to 816 deleted in pRL662, Gm^R	[6]
$pG1^{804}$	EML4568	*vgrG1* encoding VgrG1 variants with amino acid residue 805 to 816 deleted was cloned into pRL662, Gm^R	[6]
$pG1^{785}$	EML4599	*vgrG1* encoding VgrG1 variants with amino acid residue 786 to 816 deleted was cloned into pRL662, Gm^R	[6]
$pG1^{781}$	EML4598	*vgrG1* encoding VgrG1 variants with amino acid residue 782 to 816 deleted was cloned into pRL662, Gm^R	[6]
pJQ200KS-*tssB*	EML949	*tssB*-flanking sequences in pJQ200KS for generating in-frame deletion mutant, Gm^R	[5]

pJQ200KS-*vgrG1*	EML954	*vgrG1*-flanking sequences in pJQ200KS plasmid to generate in-frame deletion mutant, Gm^R	[5]
pJQ200KS-*vgrG2* operon	EML2689	*vgrG2* operon-flanking sequences in pJQ200KS plasmid to generate in-frame deletion mutant, Gm^R	[5]
pJQ200KS-*G1_785*		*vgrG1*-flanking sequences in pJQ200KS plasmid to generate truncated VgrG1_785 mutant, Gm^R	This study
pJQ200KS-*G1_781*		*vgrG1*-flanking sequences in pJQ200KS plasmid to generate truncated VgrG1_781 mutant, Gm^R	This study
pJQ200KS-*tae-tai**		*tae-tai*-flanking sequences in pJQ200KS plasmid to generate in-frame deletion mutant, Gm^R	This study
pJQ200KS-*v1* (12D1)	EML4670	*v1*-flanking sequences from 12D1 in pJQ200KS plasmid to generate in-frame deletion mutant, Gm^R	This study
pJQ200KS-*v3-4* (12D1)	EML4671	*v3-4*-flanking sequences from 12D1 in pJQ200KS plasmid to generate in-frame deletion mutant, Gm^R	This study
pJQ200KS-*tae-tai* (12D1)		*tae-tai*-flanking sequences from 12D1 in pJQ200KS plasmid to generate in-frame deletion mutant, Gm^R	This study
pJQ200KS-*v4* (1D1108)	EML4686	*v4*-flanking sequences from 1D1108 in pJQ200KS plasmid to generate in-frame deletion mutant, Gm^R	This study

pJQ200KS-*v6* (1D1108)	EML4687	*v6*-flanking sequences from 1D1108 in pJQ200KS plasmid to generate in-frame deletion mutant, Gm^R	This study
pJQ200KS-*v1-9* (1D1108)	EML4645	*v1-9*-flanking sequences from 1D1108 in pJQ200KS plasmid to generate in-frame deletion mutant, Gm^R	This study
pJQ200KS-*v1-7* (15955)	EML4644	*v1-7*-flanking sequences from 15955 in pJQ200KS plasmid to generate in-frame deletion mutant, Gm^R	This study
pDONR222		Gateway cloning Donor vector, Km^R	Invitrogen
pRL662_RfC.1		Gateway reading frame cassette C.1 (RfC.1) cloned in pRL662, Gm^R	[9]
pTrc200_RfC.1		Gateway reading frame cassette C.1 (RfC.1) cloned in pTrc200, Sp^R	[9]
pTdei2		*tde2* and *tdi2* were cloned into pTrc200, Sp^R	This study
pV1 (12D1)		*v1* from 12D1 was cloned into pRL662, Gm^R	This study
pV3-4 (12D1)		*v3-4* from 12D1 was cloned into pRL662, Gm^R	This study
pV4 (1D1108)		*v4* from 1D1108 was cloned into pRL662, Gm^R	This study
pV6 (1D1108)		*v6* from 1D1108 was cloned into pRL662, Gm^R	This study

Appendix Table S2. Primers used in this study

Name	Sequence (5’ to 3’)[a]	Related construct(s)
*tssB*_XmaI_F	CCCGGGAAACCCGGCGGAGACAATAA	pTrc-TssB-GFP
*GFP*_HindIII_R	AAGCTTCTATTTGTATAGTTCATCCATGCCA	pTrc-TssB-GFP
*tssB-GFP*_F	AAAAGAACGGCGCGAGCGAAATGAGTAAAGGAGAAGAACTTTT	pTrc-TssB-GFP
*tssB-GFP*_R	AAAAGTTCTTCTCCTTTACTCATTTCGCTCGCGCCGTTCTTTT	pTrc-TssB-GFP
*tssB*_F	TTGGATACACAGCATGTTAA	Δ*tssL*Δ*tssB*, Δ*tdei*Δ*tssB*
*tssB*_R	GTTTTCCTCCGCACAGGCGA	Δ*tssL*Δ*tssB*, Δ*tdei*Δ*tssB*
*G1*_F	ATGCGCGTTAACTTTGACAC	Δ*tdei*Δ*vgrG1*
*G1*_R	ATTATGGGTGTGTCGTTCAT	Δ*tdei*Δ*vgrG1*
*G2op*_F	GAACAGCCTGACAATCCTGT	Δ*tdei*Δ*vgrG1*Δ*vgrG2* operon
*G2op*_R	CAGTGCCTGATAGACGTTGT	Δ*tdei*Δ*vgrG1*Δ*vgrG2* operon
*XbaI_G1*_F	ATATATTCTAGAGACACCCTCTATAGCAATTA	*vgrG1_785* Δ*vgrG2* operon
*BamHI_G1*_R	ATATATGGATCCAGGTCCTAAAAACCGG	*vgrG1_785* Δ*vgrG2* operon

*785*_F	CCTGAAGATCGATATCGAA TGATTATGAACGACACACC	*vgrG1_785* Δ*vgrG2* operon
*785*_R	GGTGTGTCGTTCATAATCATT CGATATCGATCTTCAGG	*vgrG1_785* Δ*vgrG2* operon
*781*_F	GCTCTTTGCCCTGAAG TGATTATGAACGACACACC	*vgrG1_781* Δ*vgrG2* operon
*781*_R	GGTGTGTCGTTCATAATCACT TCAGGGCAAAGAGC	*vgrG1_781* Δ*vgrG2* operon
*tai*_Up_F	ATCGAATTCCTGCAGACATCG CAAGTGGATGGATATT	pJQ200KS-*tae-tai* (new)
*tai*_Up_R	ATATAGGGAGCGCATGAAA	pJQ200KS-*tae-tai* (new)
*tae*_Down_F	ATGCGCTCCCTATATGCCTGC TACTACAATTCG	pJQ200KS-*tae-tai* (new)
*tae*_Down_F	AGAACTAGTGGATCCCGAAA CGGCGAGTGAGATAA	pJQ200KS-*tae-tai* (new)
Tde2_F	CAAAAAAGCAGGCTCCTGCA AGGGAGACAGCATGAGT	pTdei2

Tdi2_R	GAAAGCTGGGTGTCACCTCGCCGAACCGATTTTCCT	pTdei2
V1_12D1_F	CAAAAAAGCAGGCTCCGAAGTAGGGGCCGACGTGGAA	pV1 (12D1)
V1_12D1_R	GAAAGCTGGGTGTCATGACGATATCCTCTCAACTGC	pV1 (12D1)
V3_12D1_EcoRI_F	ATCGAATTCCTGCAGATAGAGGAGGAGTTCGAGC	pV3-4 (12D1)
V4_12D1_BamHI_R	AGAACTAGTGGATCCGCATTTATATCTCATCGACC	pV3-4 (12D1)
V4_1D1108_F	CAAAAAAGCAGGCTCCTCTGAATTGACCAGTATGTGG	pV4 (1D1108)
V4_1D1108_R	GAAAGCTGGGTGTCATGAGACCACCCCAGCCATTTG	pV4 (1D1108)
V6_1D1108_F	CAAAAAAGCAGGCTCCCGGTGGGATAGACGCATGAAG	pV6 (1D1108)
V6_1D1108_R	GAAAGCTGGGTGCTACCAGCTTGGTATCGGAG	pV6 (1D1108)
V1_12D1_Up_F	GCGTCTAGATTGGTGCTGGCGGTATTTC	pJQ200KS-*v1* (12D1)
V1_12D1_Up_R	CTAGGATCCGCTGTTGAGTTCCACGTCG	pJQ200KS-*v1* (12D1)
V1_12D1_Down_F	TCAGGATCCGAGAGGATATCGTCATGATC	pJQ200KS-*v1* (12D1)
V1_12D1_Down_R	AAGCCCGGGTCGAACTCCTCCTCTATGTCG	pJQ200KS-*v1* (12D1)

V3-4_12D1_Up_F	GAGTCTAGAGATGAATGCGATGTTGCACG	pJQ200KS-*v3-4* (12D1)
V3-4_12D1_Up_R	GAGGGATCCGAGGTGCTGGCTCATTGAAC	pJQ200KS-*v3-4* (12D1)
V3-4_12D1_Down_F	GAAGGATCCAAGGTCGATGAGATATAAATGC	pJQ200KS-*v3-4* (12D1)
V3-4_12D1_Down_R	CTACCCGGGCTCTATCTCAGGAAAGAAGG	pJQ200KS-*v3-4* (12D1)
*tai*_12D1_Up_F	ATCGAATTCCTGCAGGAGCTTACAGCATGCCAATTTAT	pJQ200KS-*tae-tai* (12D1)
*tai*_12D1_Up_R	CGCATATAGGGAGCGCAT	pJQ200KS-*tae-tai* (12D1)
*tae*_12D1_Down_F	CGCTCCCTATATGCGGCCTGCTACTACAATTCG	pJQ200KS-*tae-tai* (12D1)
*tae*_12D1_Down_R	AGAACTAGTGGATCCCGTGGCTGAACCAGTAGAA	pJQ200KS-*tae-tai* (12D1)
V4_1D1108_Up_F	GAATCTAGAAGTCGGTGACGTGAATAAGG	pJQ200KS-*v4* (1D1108)
V4_1D1108_Up_R	TAAGGATCCGCTGACAGCCCACATACTGG	pJQ200KS-*v4* (1D1108)
V4_1D1108_Down_F	TAAGGATCCGCTGGGGTGGTCTCATGA	pJQ200KS-*v4* (1D1108)
V4_1D1108_Down_R	GATCCCGGGTGTCAACGACCGCAACAAGG	pJQ200KS-*v4* (1D1108)
V6_1D1108_Up_F	AGTTCTAGATGCCTATCACAGTGAACTGC	pJQ200KS-*v6* (1D1108)

V6_1D1108_Up_R	TGTGGATCCGATTGTTTCCTTCATGCGTC	pJQ200KS-*v6* (1D1108)
V6_1D1108_Down_F	TAAGGATCCCCGATACCAAGCTGGTAGG	pJQ200KS-*v6* (1D1108)
V6_1D1108_Down_R	GATCCCGGGTCGTCTGGTACATCAGGTGC	pJQ200KS-*v6* (1D1108)
V1-9_1D1108_Up_F	ACCATCTAGAGATAAATCGACGGAAGTTGG	pJQ200KS-*v1-9* (1D1108)
V1-9_1D1108_Up_R	GAGTGGATCCTGCTTCTCAATTCAAATCTATC	pJQ200KS-*v1-9* (1D1108)
V1-9_1D1108_Down_F	CTGAGGATCCGACCCCACTGAGGACTGATC	pJQ200KS-*v1-9* (1D1108)
V1-9_1D1108_Down_R	AGCCCGGGAGAAAGCCAACCTCTGCACCC	pJQ200KS-*v1-9* (1D1108)
V1-7_15955_Up_F	GACGATCTAGACGAAACAATCACAGCGAACG	pJQ200KS-*v1-7* (15955)
V1-7_15955_Up_R	CGGGATCCTGCTTCTCAATTCAAATCTATC	pJQ200KS-*v1-7* (15955)
V1-7_15955_Down_F	CGGGATCCGACCCCACTGAGGACTGATC	pJQ200KS-*v1-7* (15955)
V1-7_15955_Down_R	TCCCCCCGGGCAACATGGATCACGTCAAAC	pJQ200KS-*v1-7* (15955)

[a]Restriction enzyme sites are underlined.

References

1. Ouahrani-Bettache S, Porte F, Teyssier J, Liautard J-P, Köhler S (1999) pBBR1-GFP: A Broad-Host-Range Vector for Prokaryotic Promoter Studies. *BioTechniques* **26:** 620-622

2. Quandt J, Hynes MF (1993) Versatile suicide vectors which allow direct selection for gene replacement in gram-negative bacteria. *Gene* **127:** 15-21

3. Ma L-S, Lin J-S, Lai E-M (2009) An IcmF Family Protein, ImpLM, Is an Integral Inner Membrane Protein Interacting with ImpKL, and Its Walker A Motif Is Required for Type VI Secretion System-Mediated Hcp Secretion in Agrobacterium tumefaciens. *Journal of Bacteriology* **191:** 4316-4329

4. Ma L-S, Hachani A, Lin J-S, Filloux A, Lai E-M (2014) Agrobacterium tumefaciens Deploys a Superfamily of Type VI Secretion DNase Effectors as Weapons for Interbacterial Competition In Planta. *Cell host & microbe* **16:** 94-104

5. Lin J-S, Ma L-S, Lai E-M (2013) Systematic Dissection of the Agrobacterium Type VI Secretion System Reveals Machinery and Secreted Components for Subcomplex Formation. *PLoS ONE* **8:** e67647

6. Bondage DD, Lin J-S, Ma L-S, Kuo C-H, Lai E-M (2016) VgrG C terminus confers the type VI effector transport specificity and is required for binding with PAAR and adaptor–effector complex. *Proceedings of the National Academy of Sciences of the United States of America* **113:** E3931-E3940

7. Vergunst AC, Schrammeijer B, den Dulk-Ras A, de Vlaam CM, Regensburg-Tuink TJ, Hooykaas PJ (2000) VirB/D4-dependent protein translocation from Agrobacterium into plant cells. *Science* **290:** 979-982

8. Schmidt-Eisenlohr H, Domke N, Baron C (1999) TraC of IncN plasmid pKM101 associates with membranes and extracellular high-molecular-weight structures in Escherichia coli. *J Bacteriol* **181:** 5563-5571

9. Wu CF, Santos MNM, Cho ST, Chang HH, Tsai YM, Smith DA, Kuo CH, Chang J, Lai EM (2019) Plant pathogenic Agrobacterium tumefaciens strains have diverse type VI effector-immunity pairs and vary in in planta competitiveness. *Molecular plant-microbe interactions : MPMI*, 10.1094/mpmi-01-19-0021-r
