# Supplementary material for: Effector loading onto the VgrG carrier activates type VI secretion system assembly
Source: EMBO Rep. 2019 Dec 5;21(1):e47961. doi: 10.15252/embr.201947961 (PMC6945064; doi:10.15252/embr.201947961)
Supplement: Supplementary file 2 — Expanded View Figures PDF [file EMBR-21-e47961-s002.pdf]

Expanded View Figures

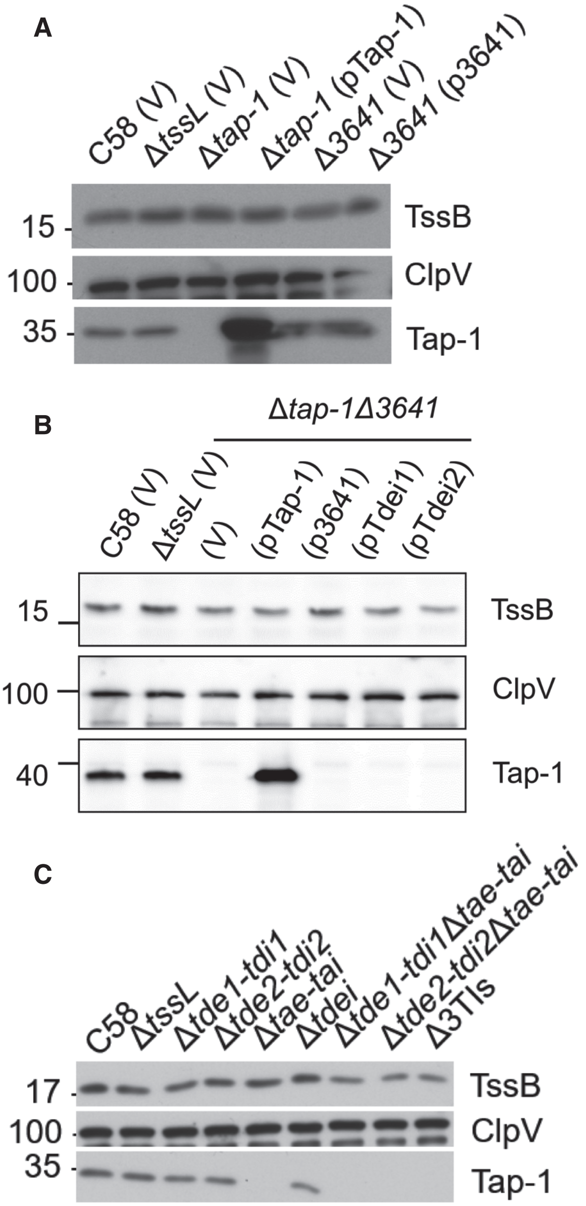

Figure EV1. Western blots of cellular fractions of representative T6SS proteins from Fig 2.

A Aliquots of the same cellular fractions that were analyzed in Fig 2A were probed with a set of different antibodies.

B Aliquots of the same cellular fractions that were analyzed in Fig 2B were probed with a set of different antibodies.

C Aliquots of the same cellular fractions that were analyzed in Fig 1B were probed with a set of different antibodies.

Data information: In (A–C), Western blots were probed with antibodies against TssB (representative protein encoded by *imp* operon), ClpV (representative protein encoded by *hcp* operon), and Tap-1. Molecular weight markers (in kDa) are indicated on the left. Data are from one independent experiment and reproduced in at least two independent experiments.
Source data are available online for this figure.

© 2019 The Authors

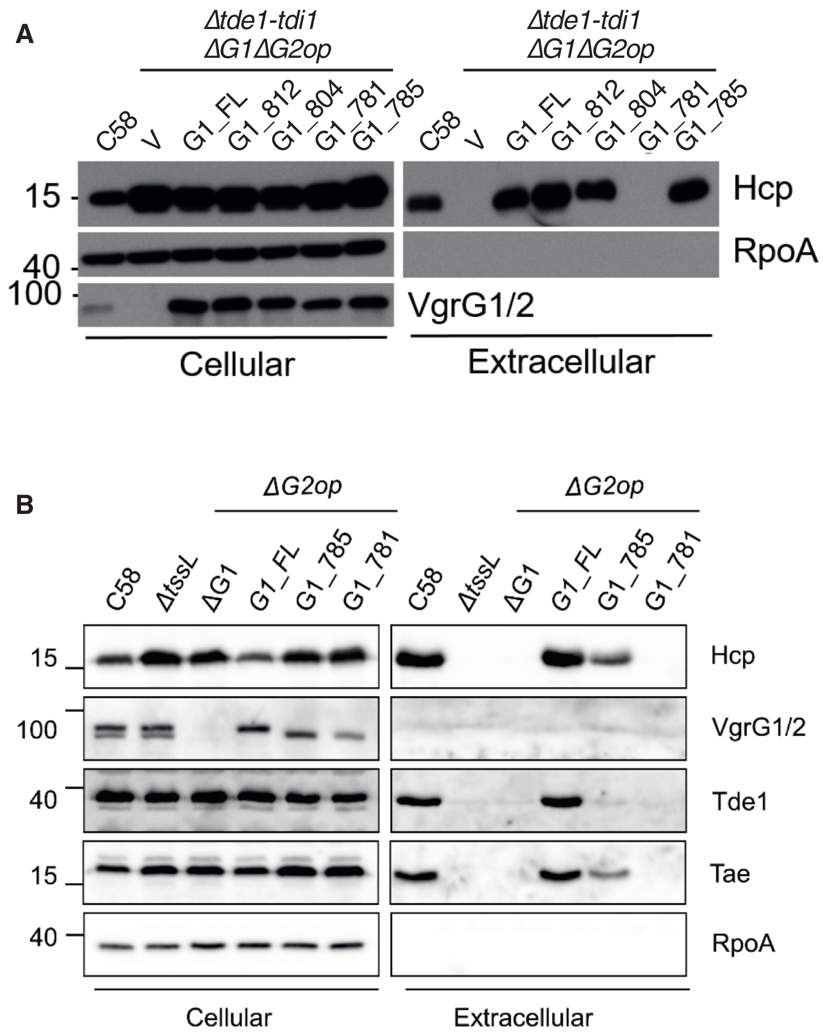

Figure EV2. Expression levels of truncated VgrG1 variants affected Hcp secretion.

A T6SS secretion assay of *Agrobacterium tumefaciens* C58 *ΔtdeiΔvgrG1ΔvgrG2* (*ΔtdeiΔG1ΔG2op* mutant) harboring a pRL662 vector (V) or its derivatives overexpressing full-length and truncated VgrG1 proteins.

B T6SS secretion assay of *A. tumefaciens* C58*ΔG2op* mutant encoding full-length and truncated VgrG1 proteins.

Data information: In (A, B), *A. tumefaciens* cells were grown in I-medium (pH 5.5) and cellular and extracellular fractions were collected for Western blot analysis probed with antibodies for indicated proteins. Molecular weight markers (in kDa) are indicated on the left. Data are from one independent experiment and reproduced in at least two independent experiments.
Source data are available online for this figure.

 © 2019 The Authors

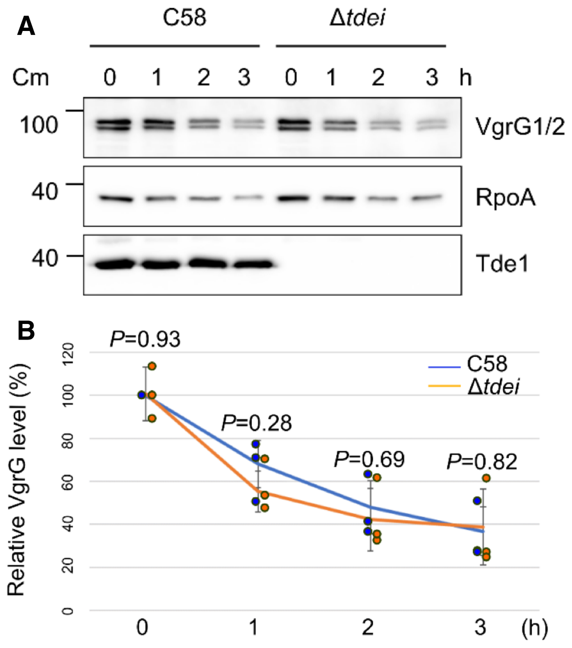

Figure EV3. VgrG protein stability is not affected by the absence of Tde-Tdi pairs.

A At times indicated, total protein fractions were collected from wild-type C58 or Δ*tdei* grown in 523 medium with chloramphenicol and analyzed in Western blots probed with antibodies for indicated proteins.

B The intensities of bands corresponding to VgrG were quantified using ImageJ software. Intensity values were normalized to those of VgrG of wild-type C58 at 0 h. Data are mean ± SD of three independent experiments. *P* values were calculated using unpaired two-sided *t*-test, and no significant difference was detected between C58 and Δ*tdei* as indicated (P value > 0.1).

Source data are available online for this figure.

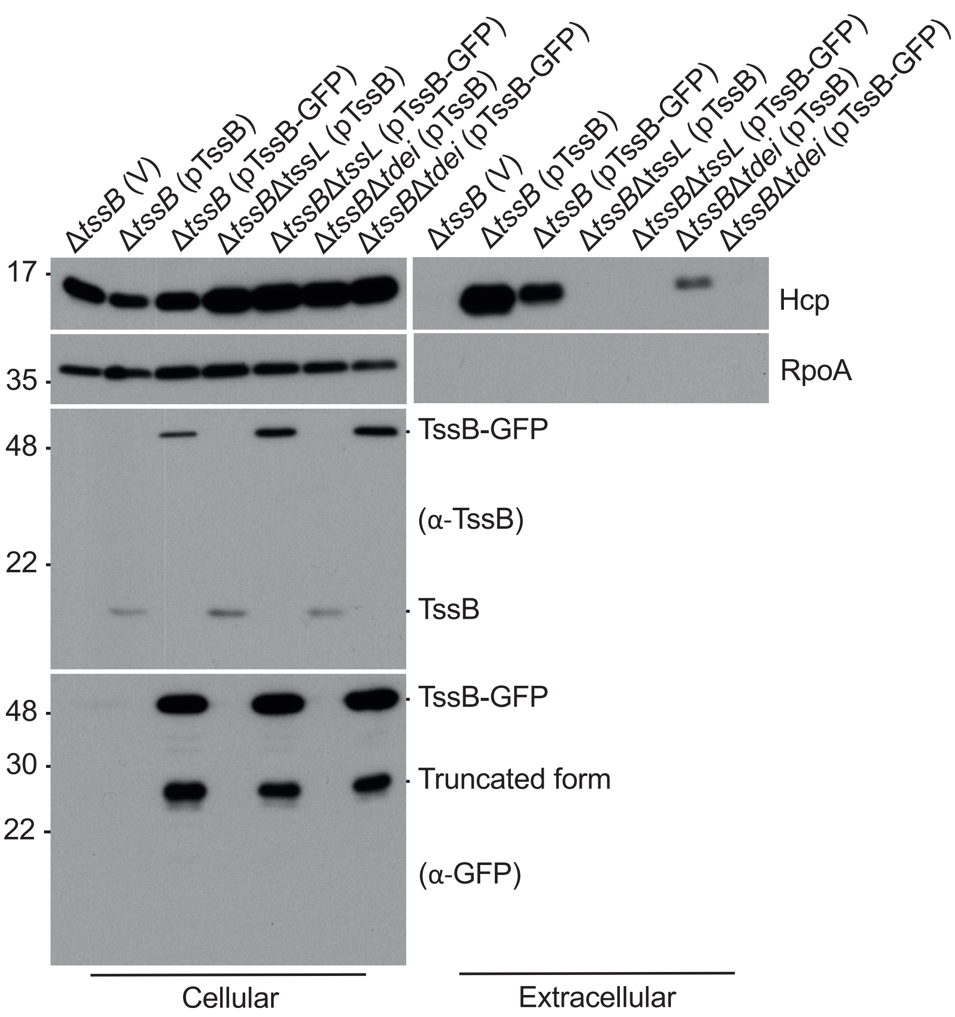

Figure EV4. C-terminal GFP-tagged TssB partially complemented Hcp secretion in Δ*tssB*.

Various *Agrobacterium tumefaciens* strains were grown in I-medium (pH5.5), and the cellular and extracellular (S) fractions were collected for Western blotting probed with antibodies for indicated proteins. Molecular weight markers (in kDa) are indicated on the left. Data are from one independent experiment and reproduced in at least two independent experiments.

Source data are available online for this figure.

 © 2019 The Authors

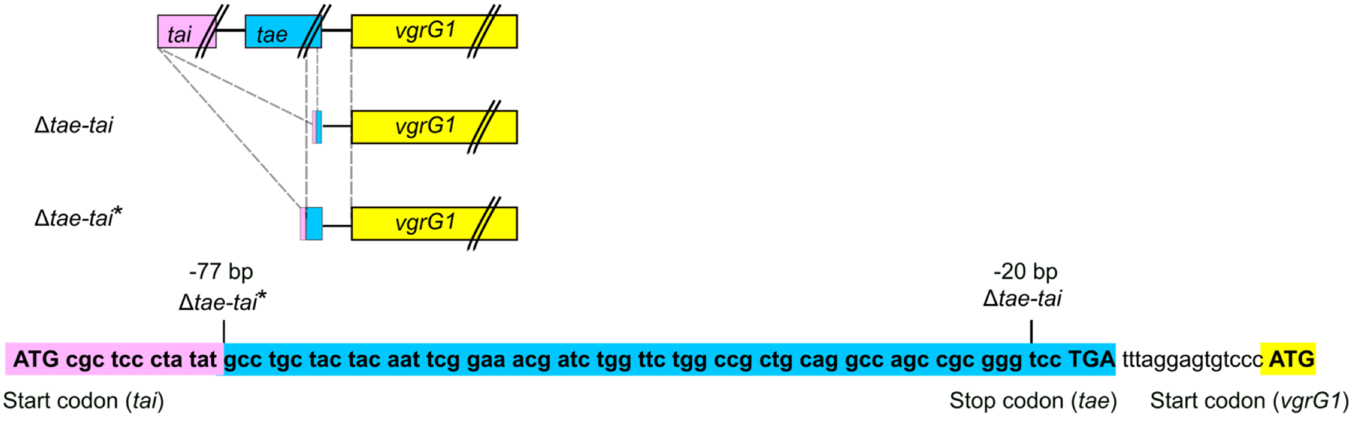

Figure EV5. Design of the non-polar *tae-tai* deletion mutation in C58.

The C-terminal coding portion of the *tai-tae* locus and N-terminal coding portion of *vgrG1* (*vgrG*) are presented. A deletion mutant of *tae-tai* was constructed to retain 77 nucleotides upstream of the start codon of the *vgrG1* coding sequence (highlighted in blue). This is predicted to generate an in-frame deletion of *tai-tae*. The original *tae-tai* deletion retained only 20 nucleotides upstream of the start codon of the *vgrG1* coding sequence.

© 2019 The Authors
