# Supplementary figures and images for: Effector loading onto the VgrG carrier activates type VI secretion system assembly
Source: EMBO Rep. 2019 Dec 5;21(1):e47961. doi: 10.15252/embr.201947961 (PMC6945064; doi:10.15252/embr.201947961)

Figure EV1A

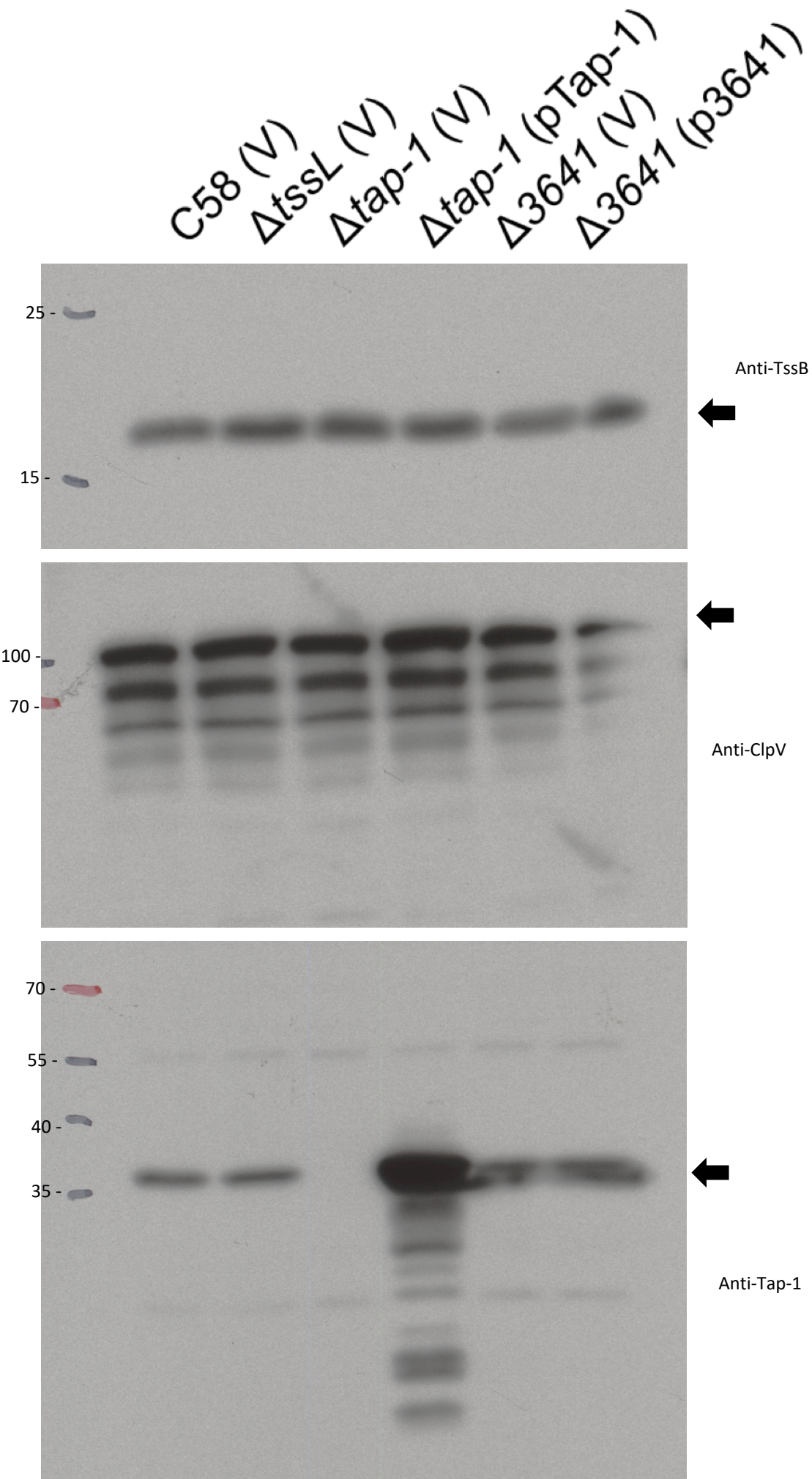

Source data for Figure EV1B

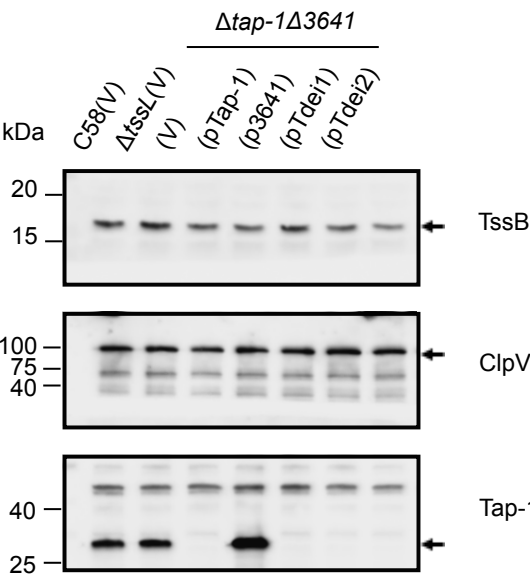

### Fig. EV1C source data

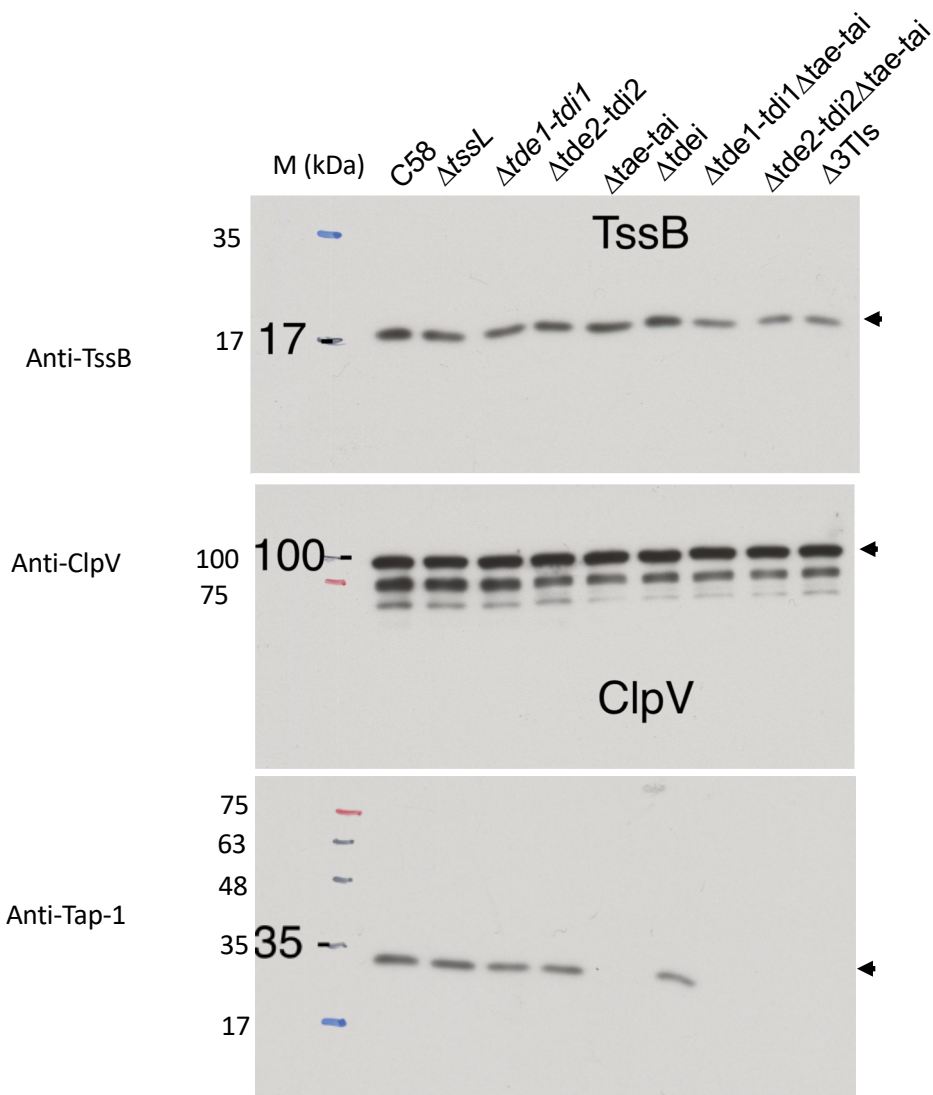

Supplement: Supplementary file 3 — Source Data for Expanded View Figures [file EMBR-21-e47961-s009.zip › Source_Data_for_EV_Figures/Source_Data_for_FigEV1.pdf]

Fig. EV2A source data

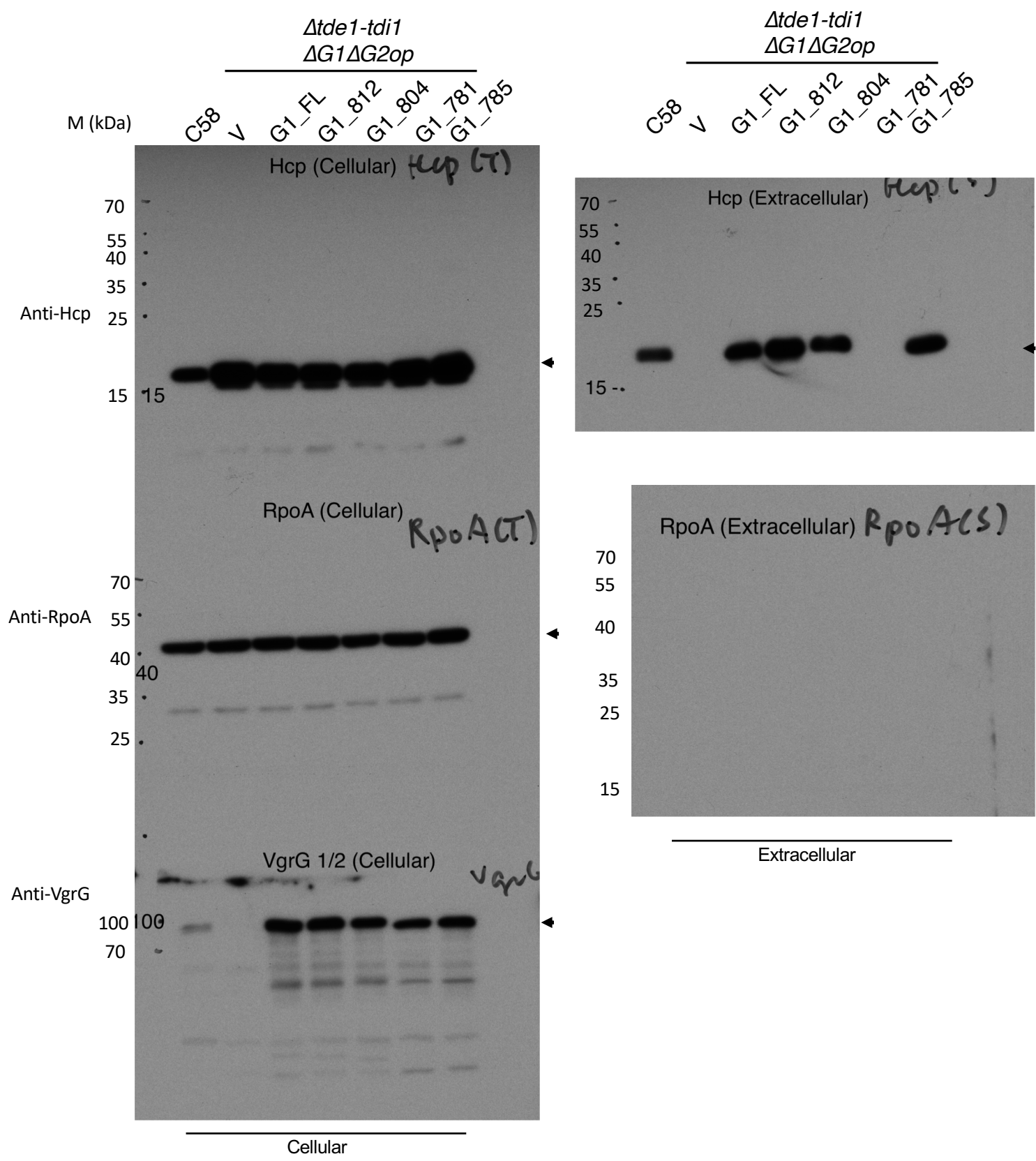

# Source data for Figure EV2B

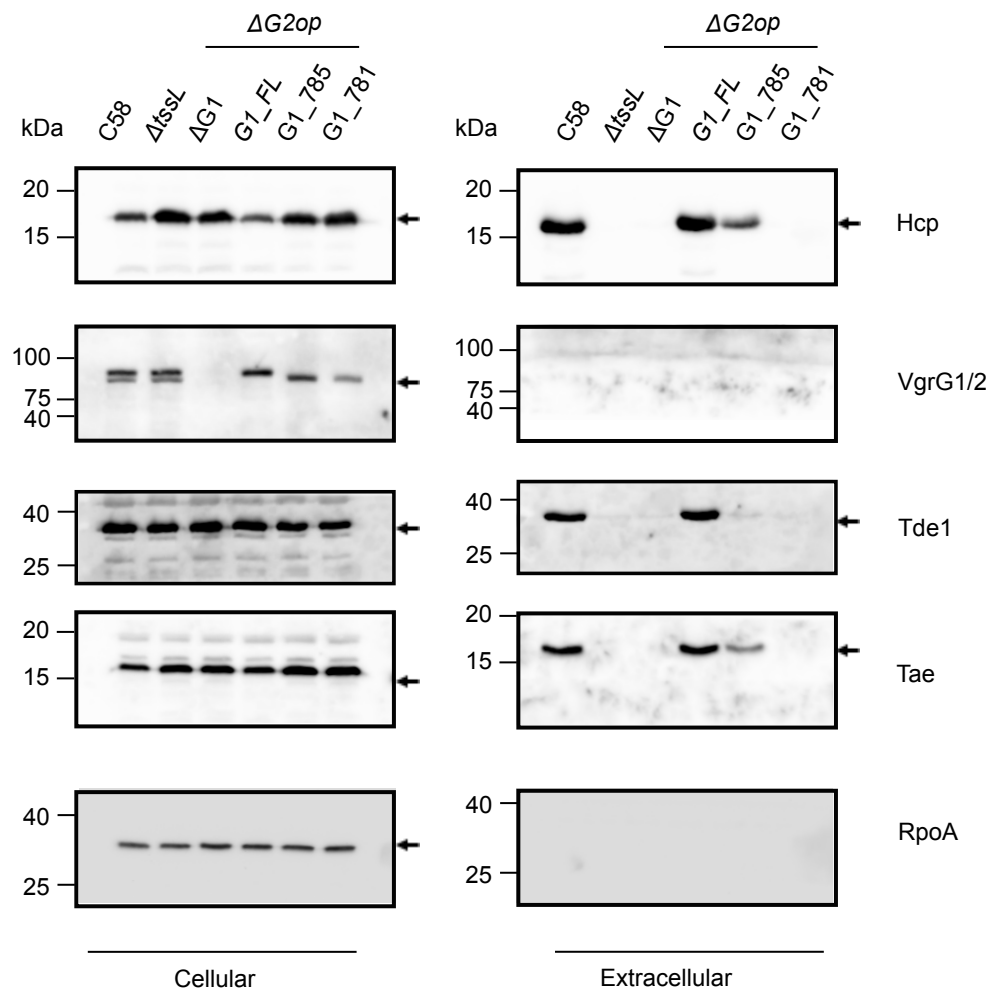

Supplement: Supplementary file 3 — Source Data for Expanded View Figures [file EMBR-21-e47961-s009.zip › Source_Data_for_EV_Figures/Source_Data_for_FigEV2.pdf]

# Source data for Figure EV3A

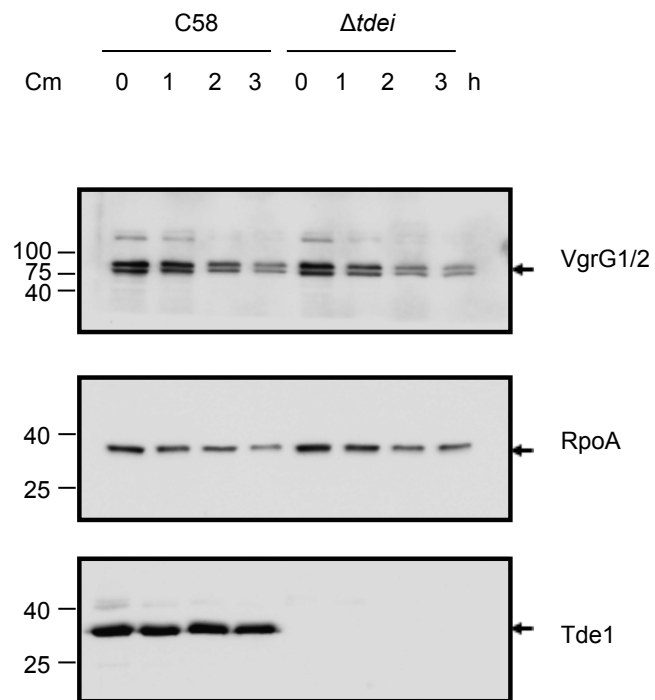

Supplement: Supplementary file 3 — Source Data for Expanded View Figures [file EMBR-21-e47961-s009.zip › Source_Data_for_EV_Figures/Source_Data_for_FigEV3.pdf]

**Fig. EV4 source data**

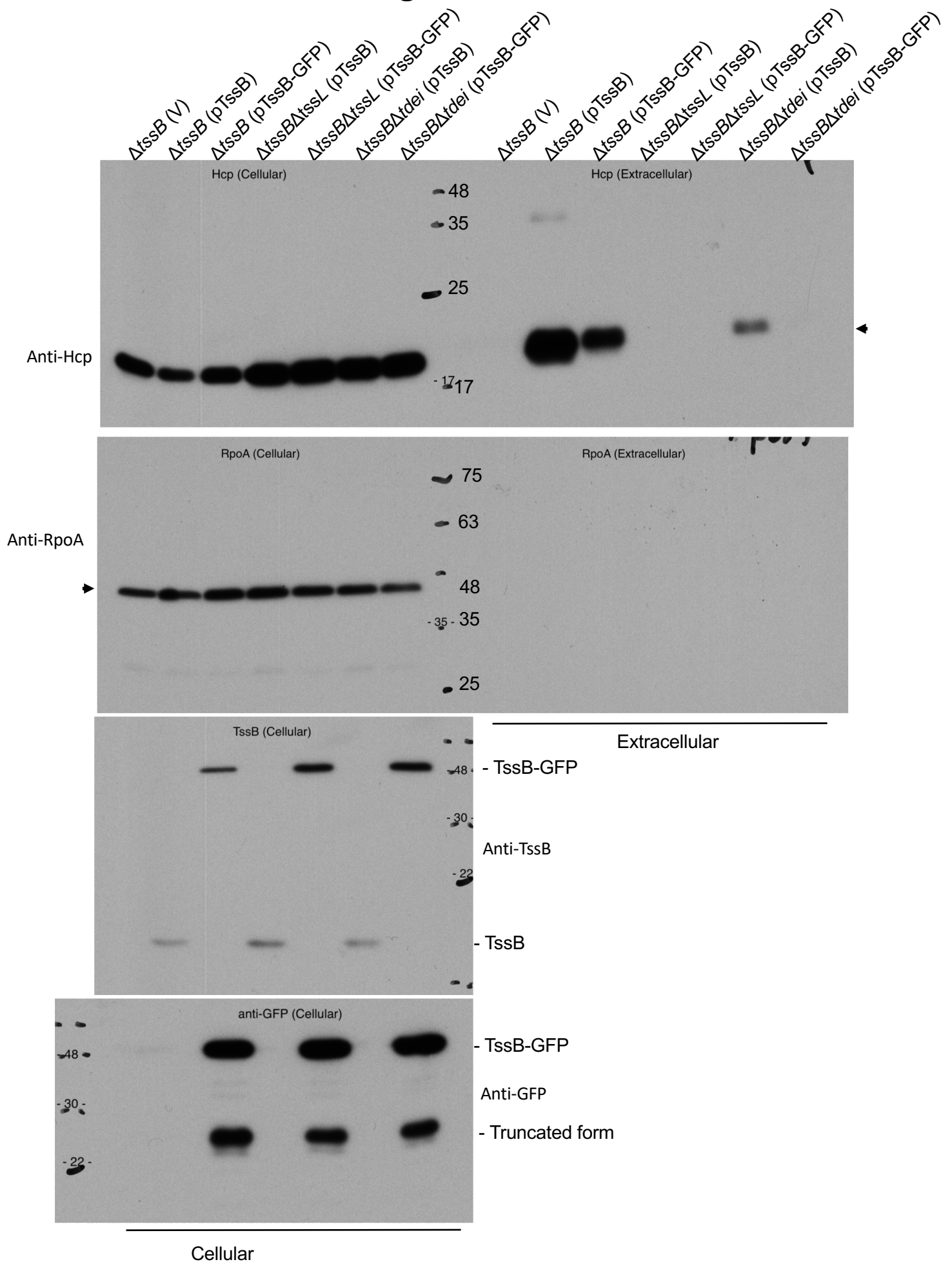

Supplement: Supplementary file 3 — Source Data for Expanded View Figures [file EMBR-21-e47961-s009.zip › Source_Data_for_EV_Figures/Source_Data_for_FigEV4.pdf]

Fig. 1B source data

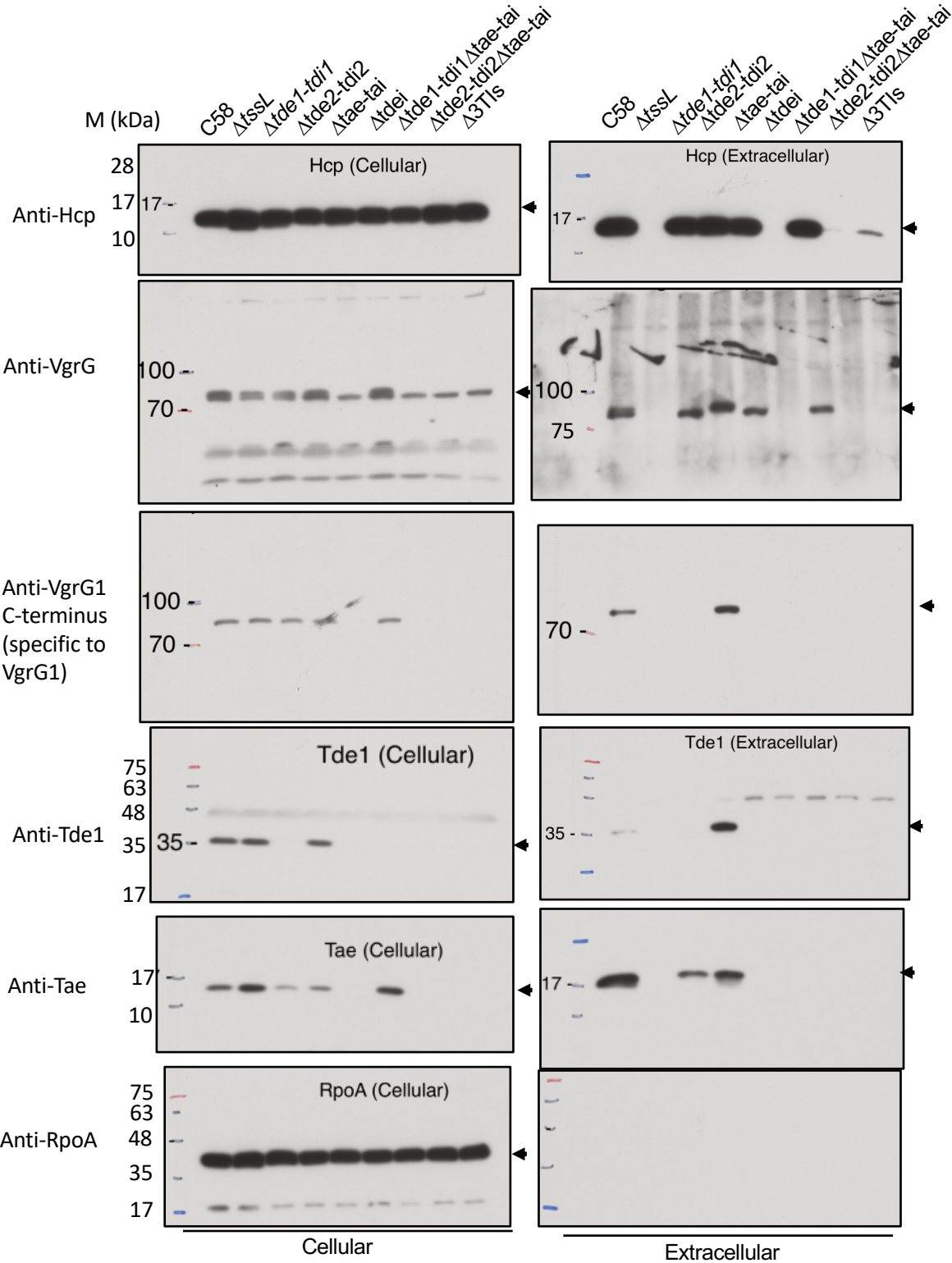

**Fig. 1C source data**

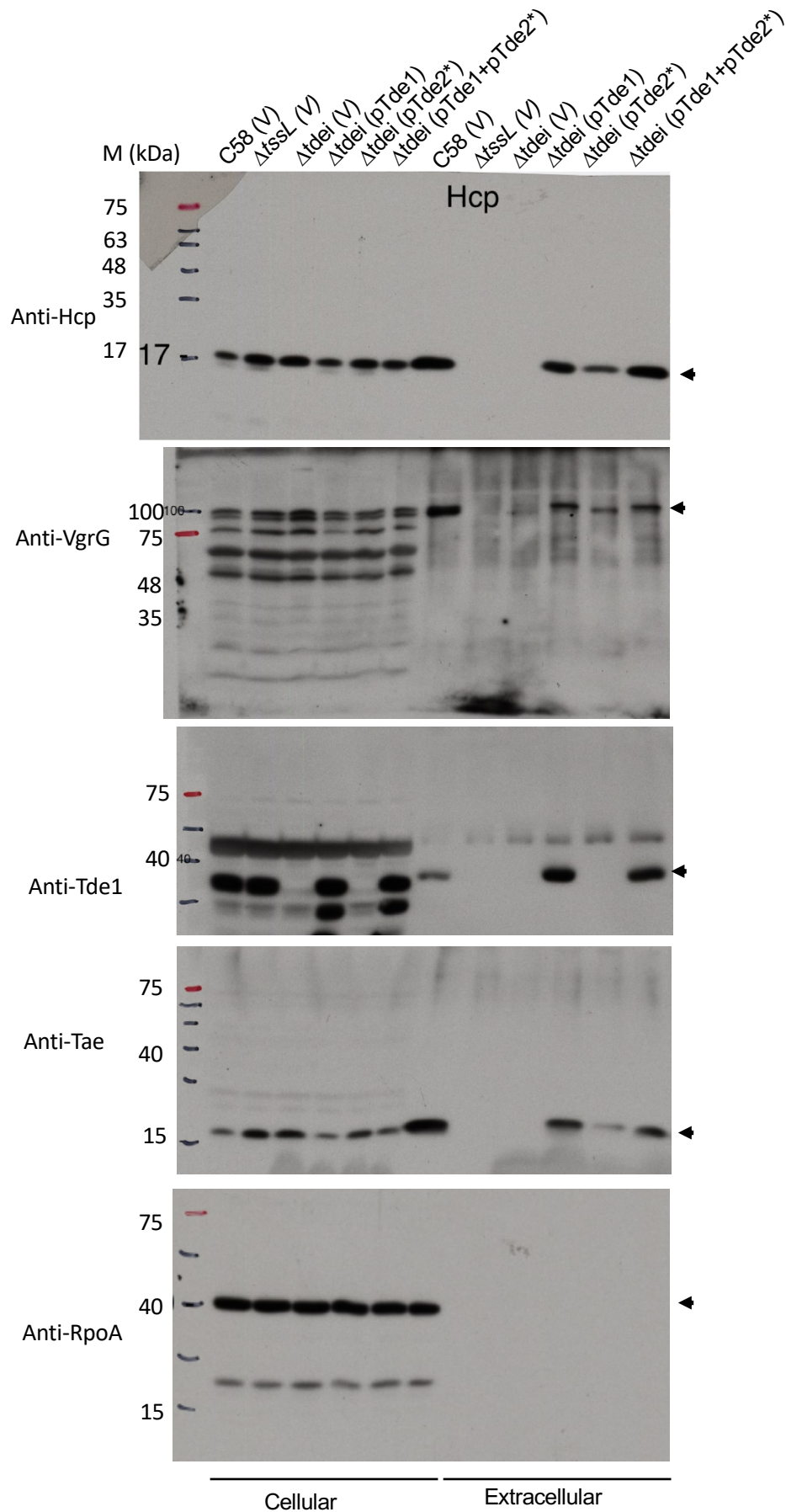

Supplement: Supplementary file 5 — Source Data for Figure 1 [file EMBR-21-e47961-s003.pdf]

### Source data for Figure 4B

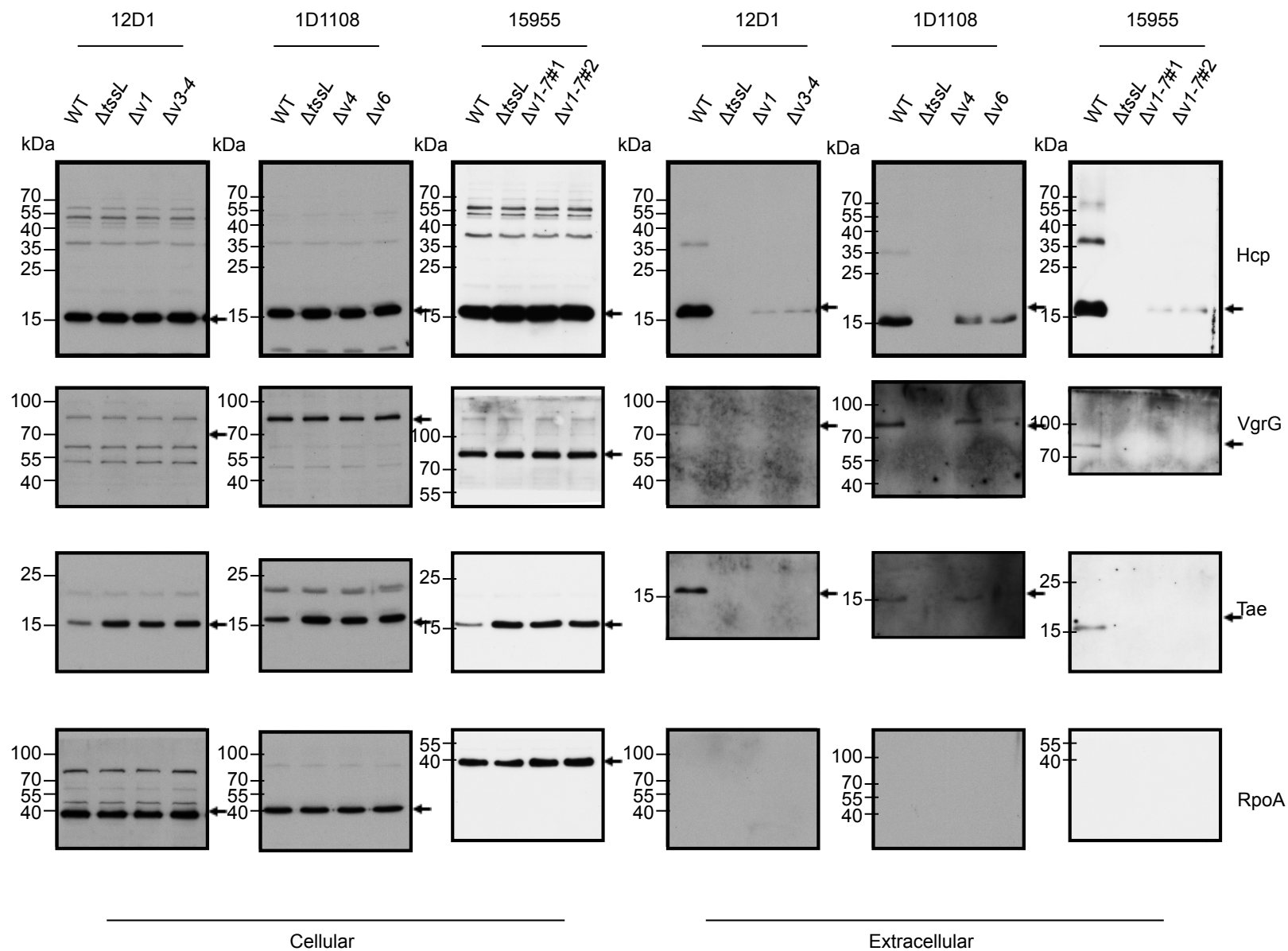

Source data for Figure 4D

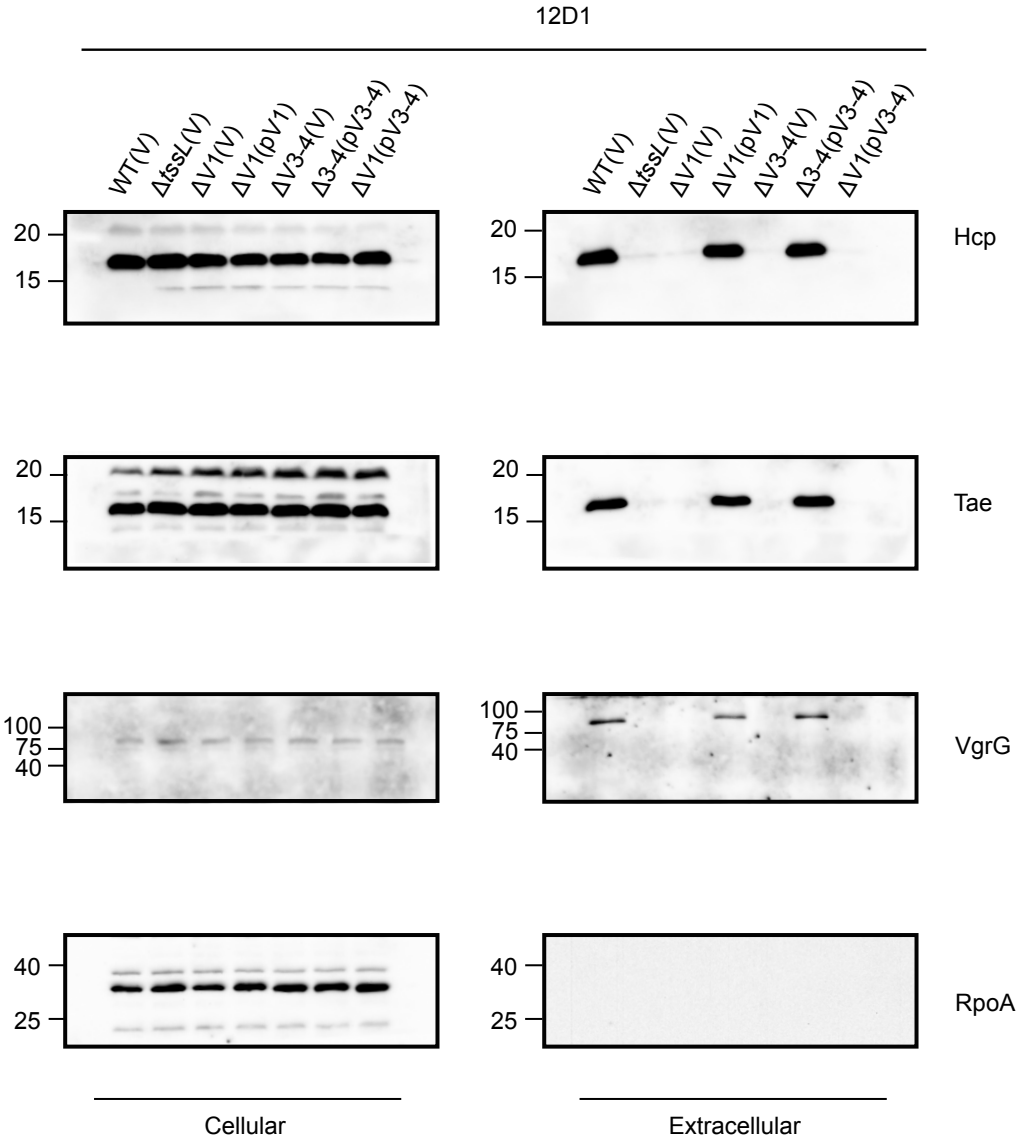

Source Data for Figure 4E

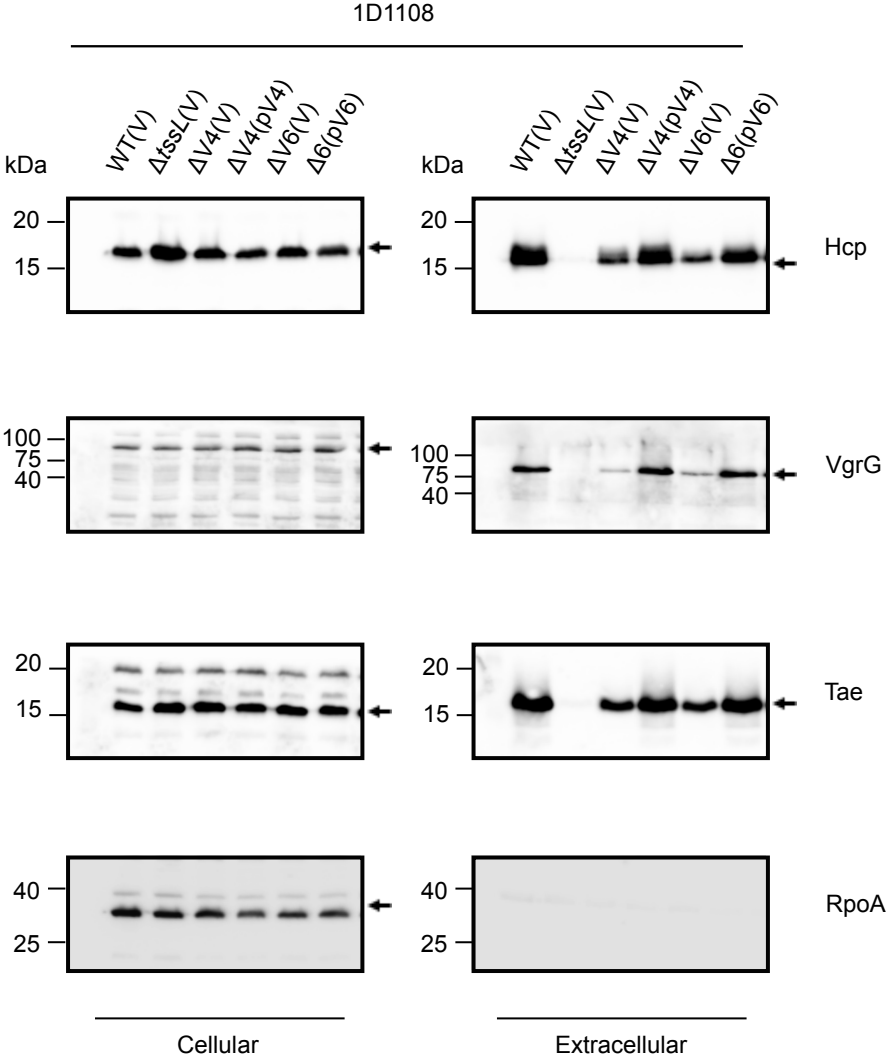

Supplement: Supplementary file 8 — Source Data for Figure 4 [file EMBR-21-e47961-s006.pdf]

Fig. 5A source data

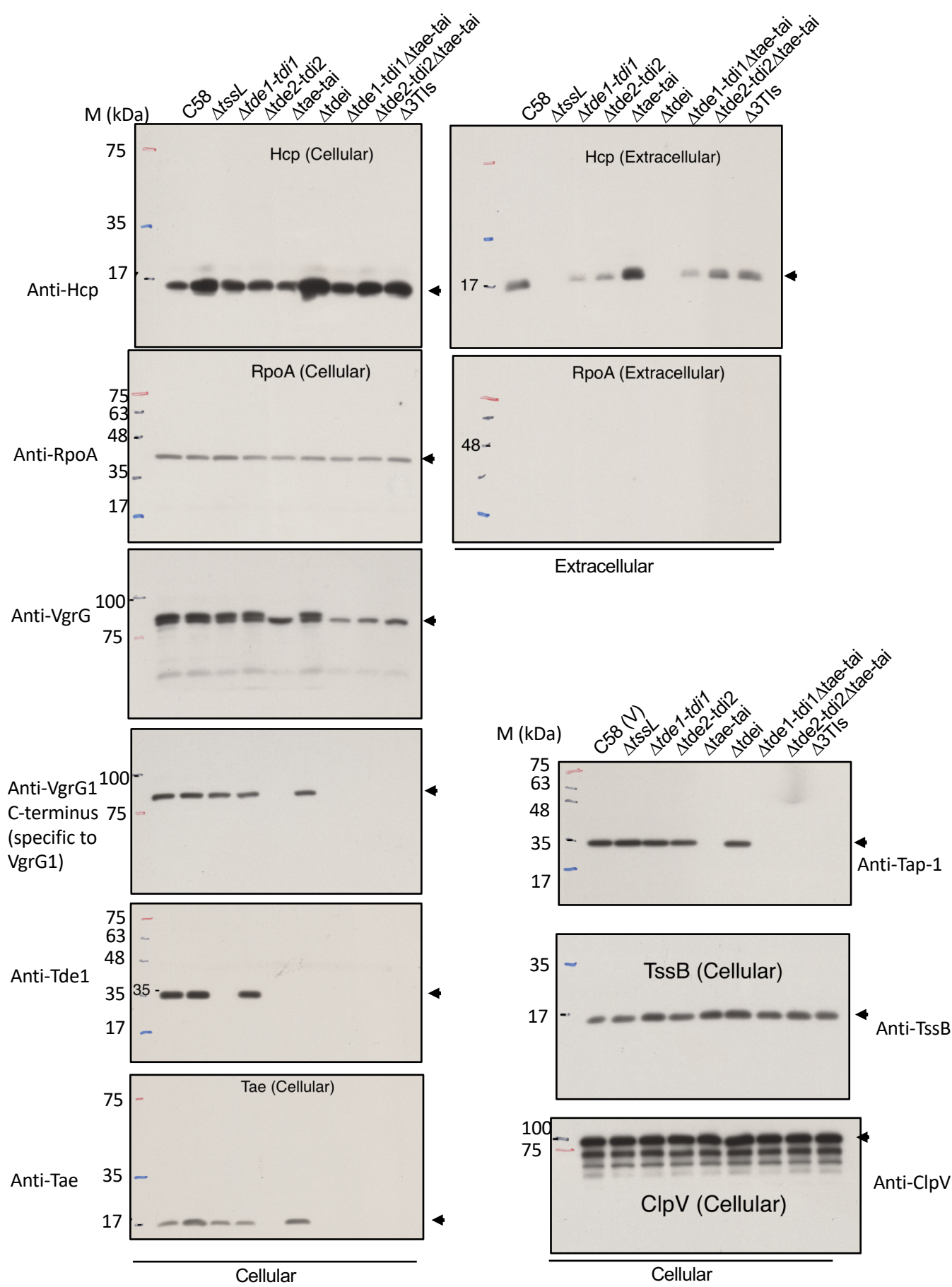

Fig. 5B source data

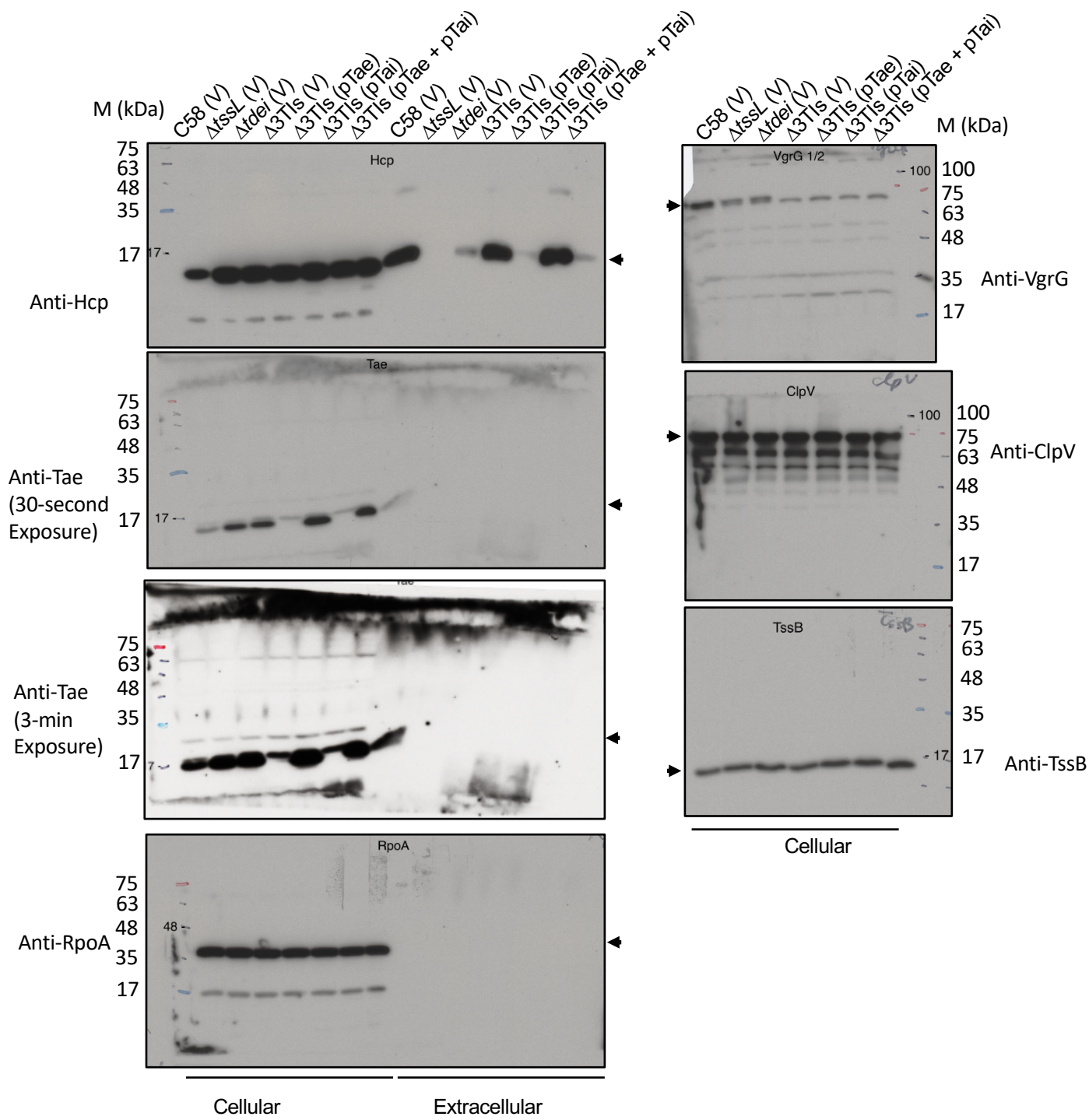

Supplement: Supplementary file 9 — Source Data for Figure 5 [file EMBR-21-e47961-s007.pdf]

# Source data for Figure 6A

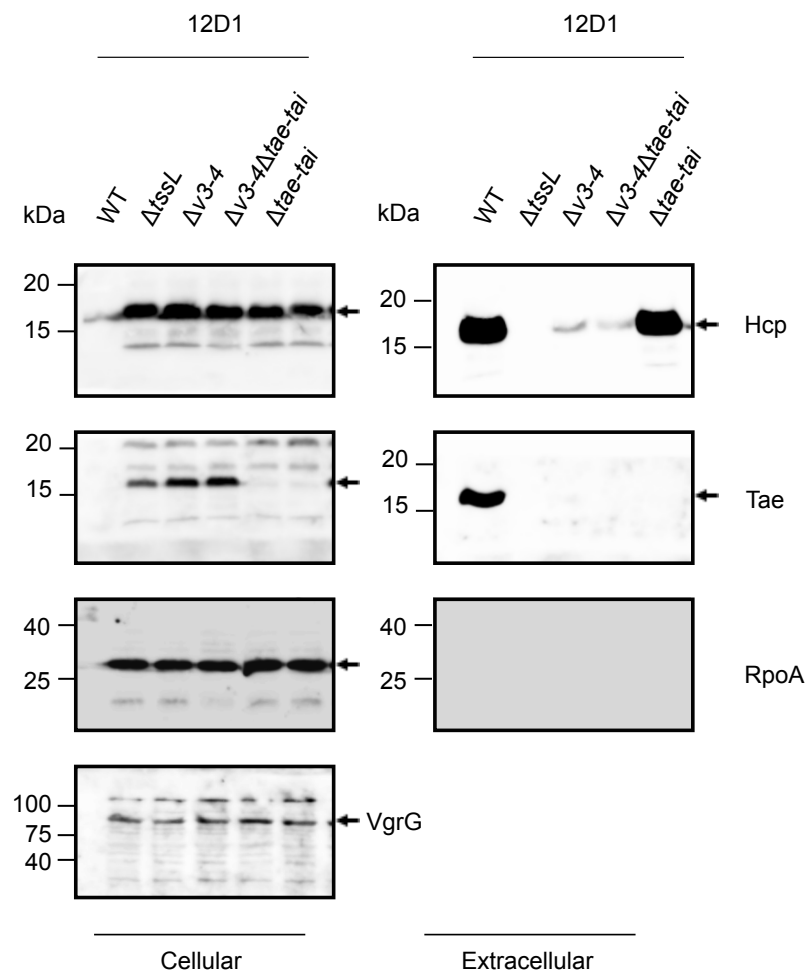

Source data for Figure 6B

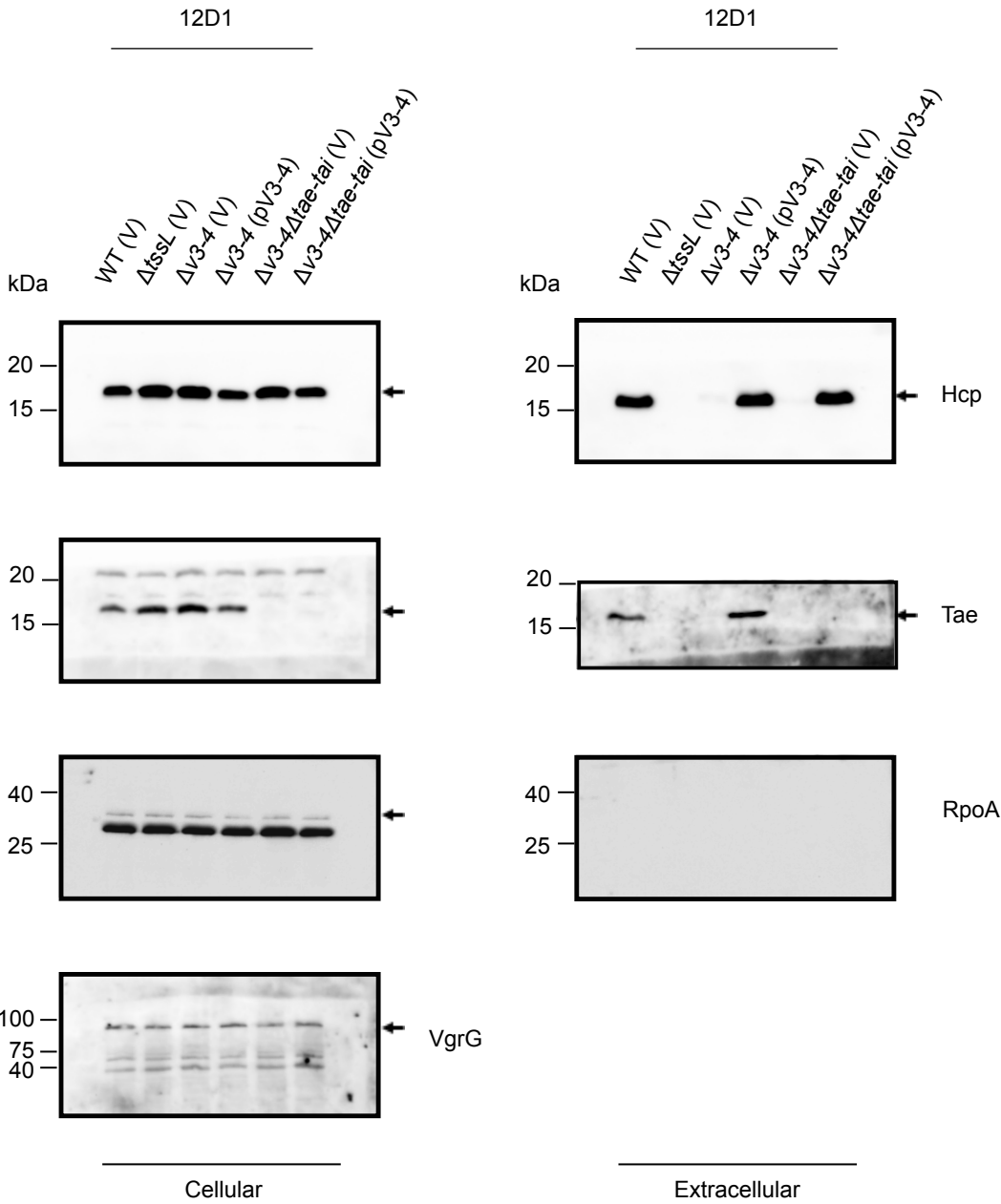

Source data for Figure 6D

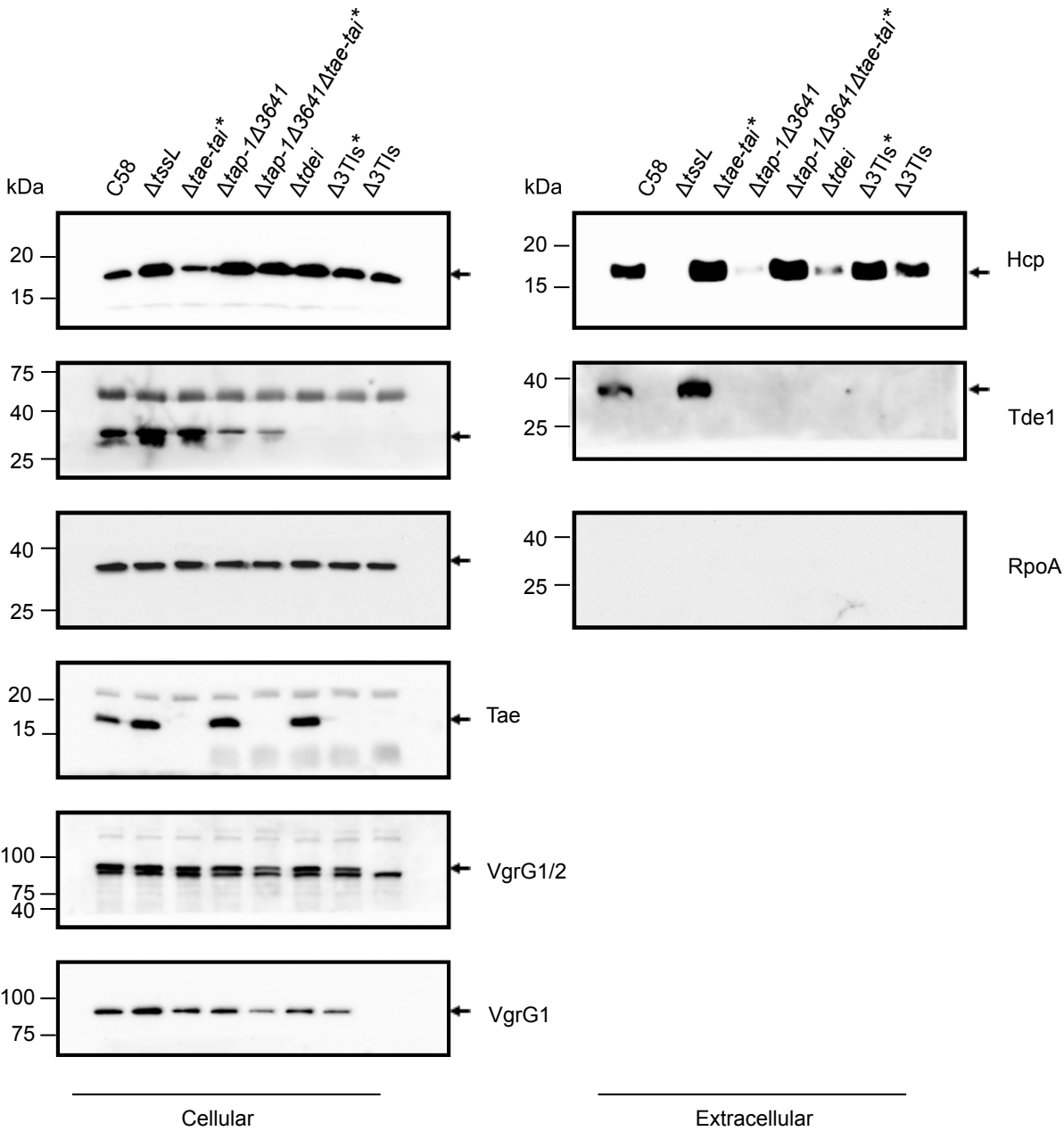

Supplement: Supplementary file 10 — Source Data for Figure 6 [file EMBR-21-e47961-s008.pdf]
