# Supplementary material for: Effector loading onto the VgrG carrier activates type VI secretion system assembly
Source: EMBO Rep. 2019 Dec 5;21(1):e47961. doi: 10.15252/embr.201947961 (PMC6945064; doi:10.15252/embr.201947961)

Figure 2A

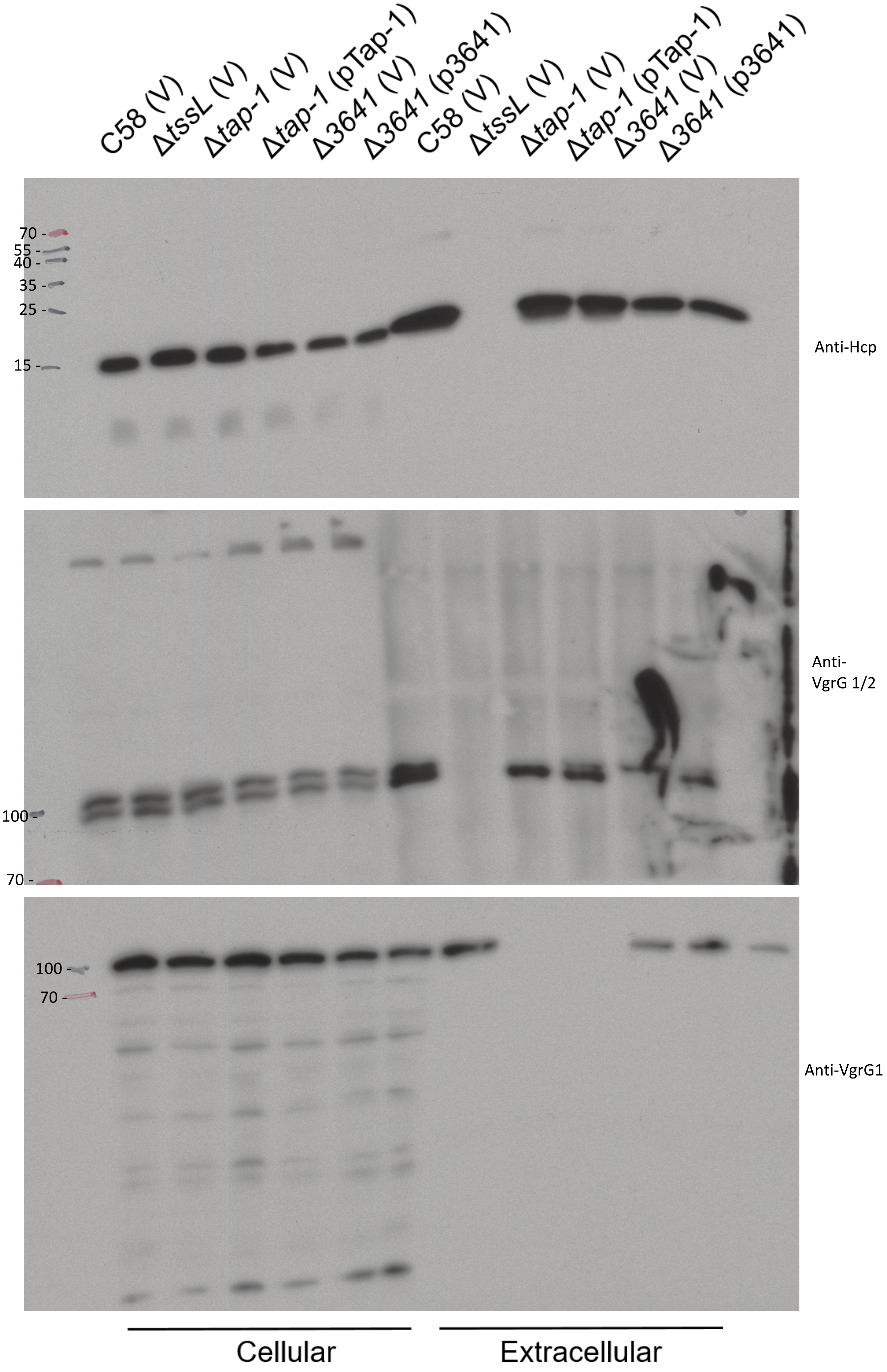
C58 (V)
ΔtssL (V)
Δtap-1 (V)
Δtap-1 (pTap-1)
Δ3641 (V)
Δ3641 (p3641)
C58 (V)
ΔtssL (V)
Δtap-1 (V)
Δtap-1 (pTap-1)
Δ3641 (V)
Δ3641 (p3641)
70 -
55 -
40 -
35 -
25 -
15 -
Anti-Hcp
100 -
70 -
Anti-
VgrG 1/2
100 -
70 -
Anti-VgrG1
Cellular
Extracellular

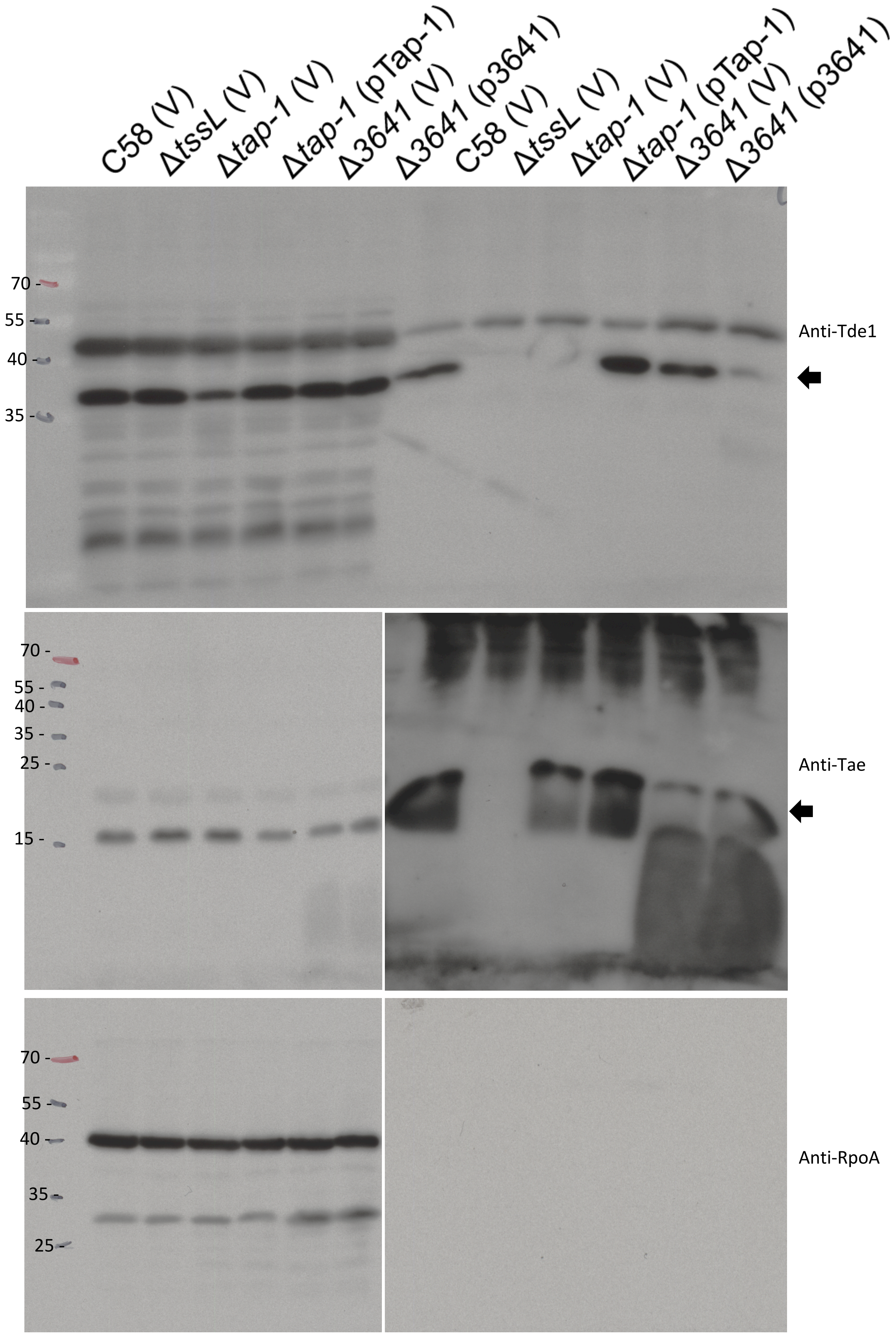
C58 (V)
ΔtssL (V)
Δtap-1 (V)
Δtap-1 (pTap-1)
Δ3641 (V)
Δ3641 (p3641)
C58 (V)
ΔtssL (V)
Δtap-1 (V)
Δtap-1 (pTap-1)
Δ3641 (V)
Δ3641 (p3641)
70 -
55 -
40 -
35 -
Anti-Tde1
70 -
55 -
40 -
35 -
25 -
15 -
Anti-Tae
70 -
55 -
40 -
35 -
25 -
Anti-RpoA

Source data for Figure 2B

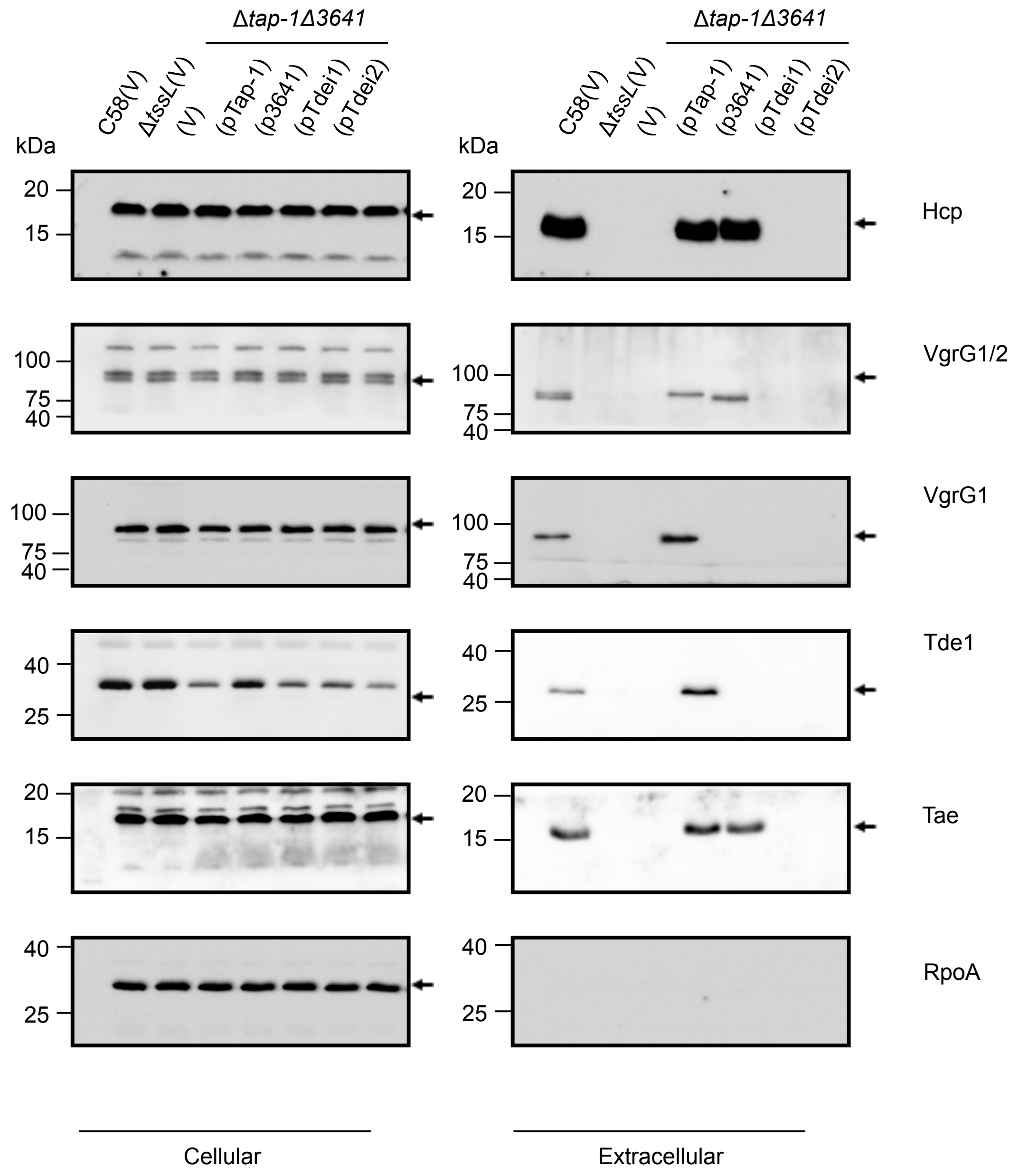

Source data for Figure 2C

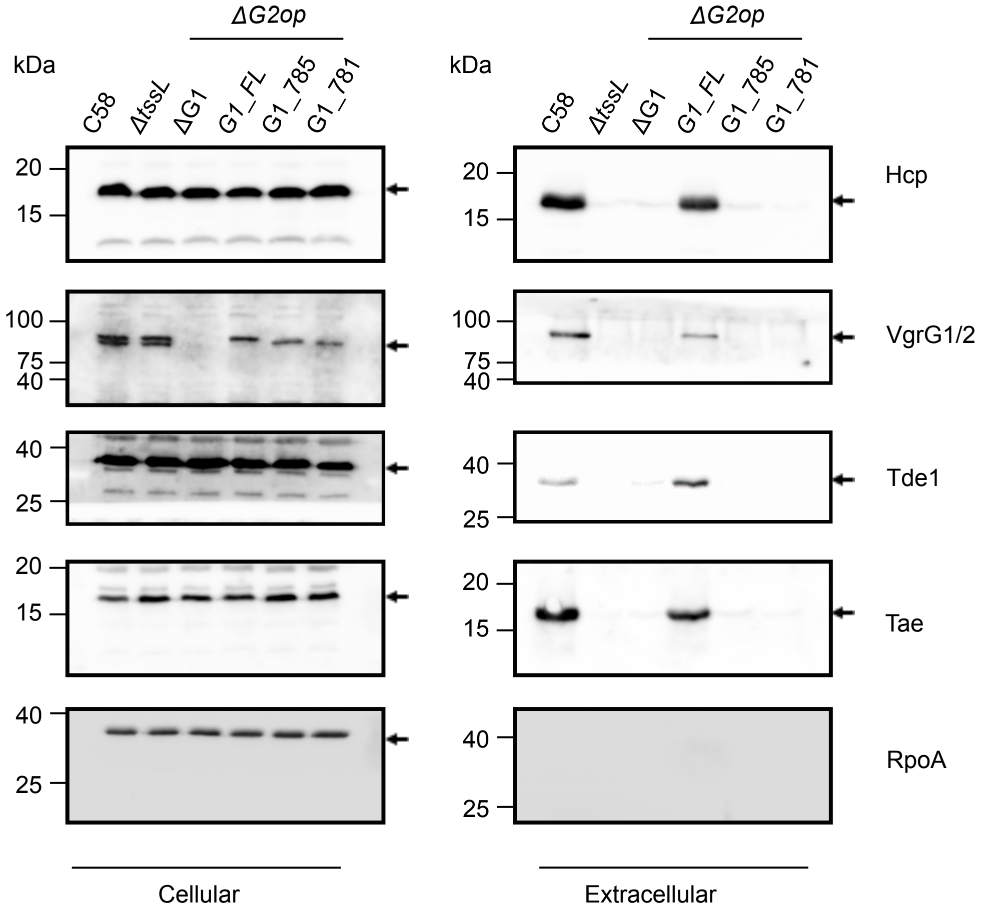

Supplement: Supplementary file 6 — Source Data for Figure 2 [file EMBR-21-e47961-s004.pdf]
