# Supplementary material for: Effector loading onto the VgrG carrier activates type VI secretion system assembly
Source: EMBO Rep. 2019 Dec 5;21(1):e47961. doi: 10.15252/embr.201947961 (PMC6945064; doi:10.15252/embr.201947961)

ΔtssB (TssB-GFP), phase contrast

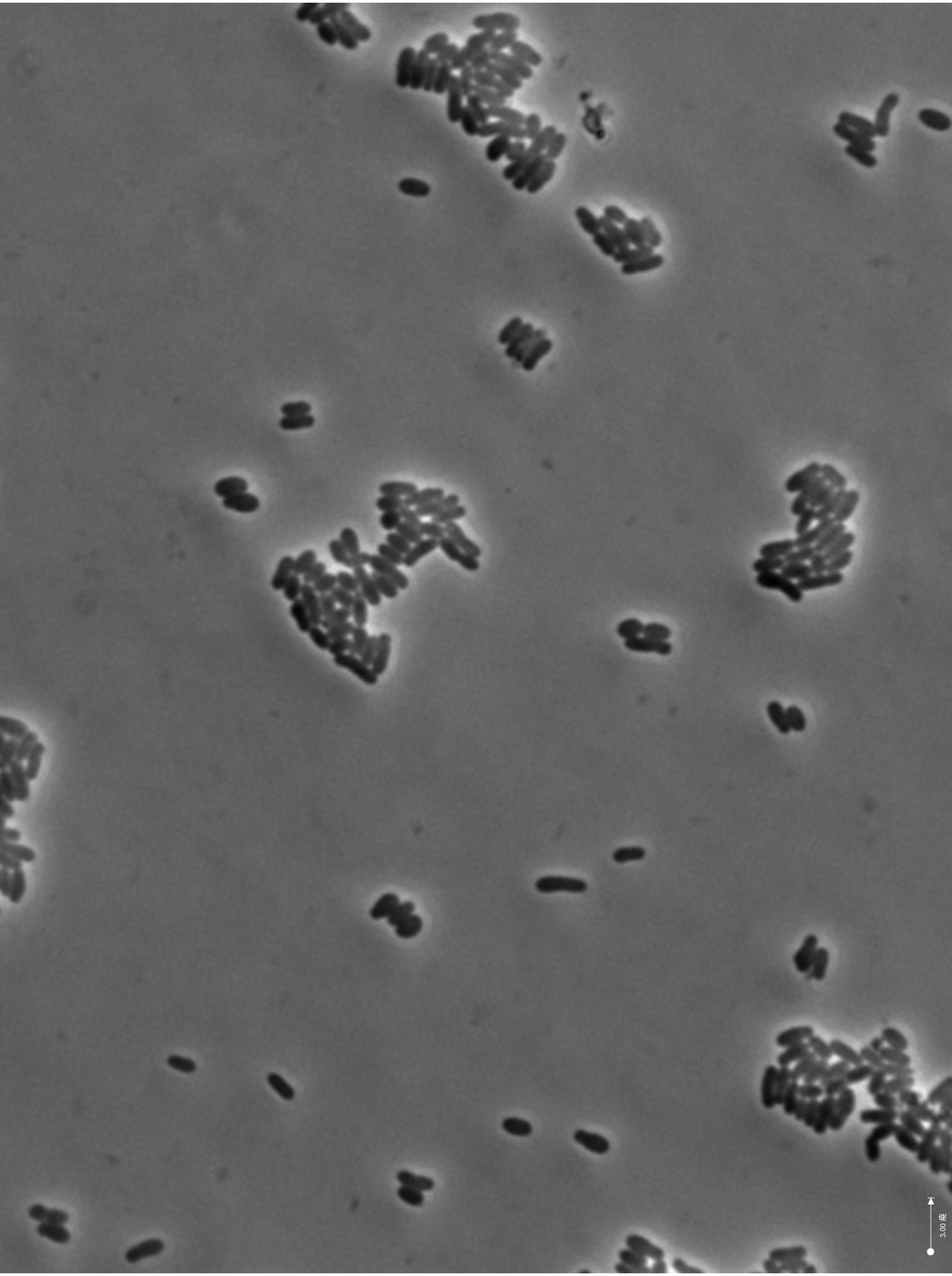

ΔtssB (TssB-GFP), GFP

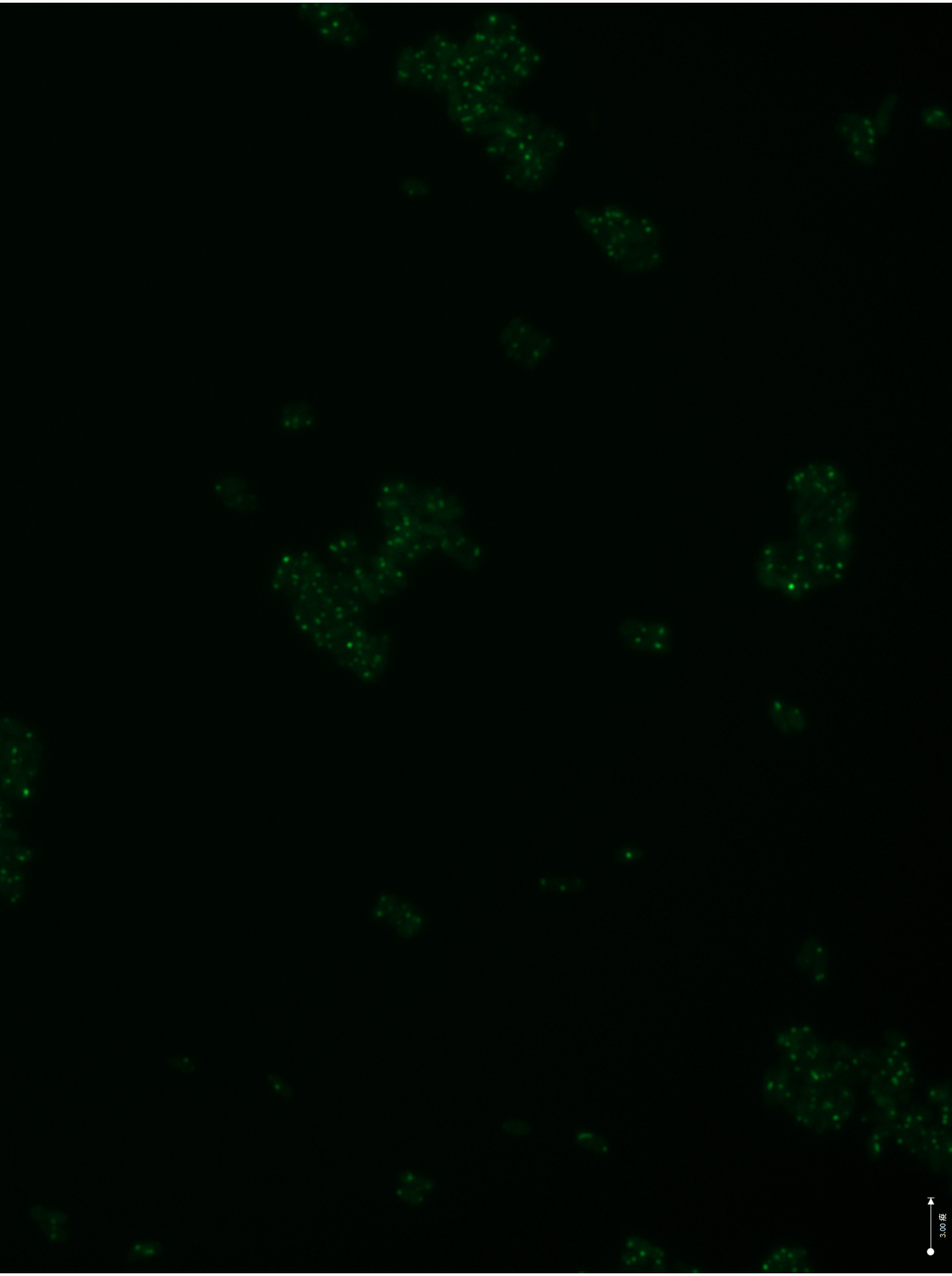

ΔtssLΔtssB (TssB-GFP), phase contrast

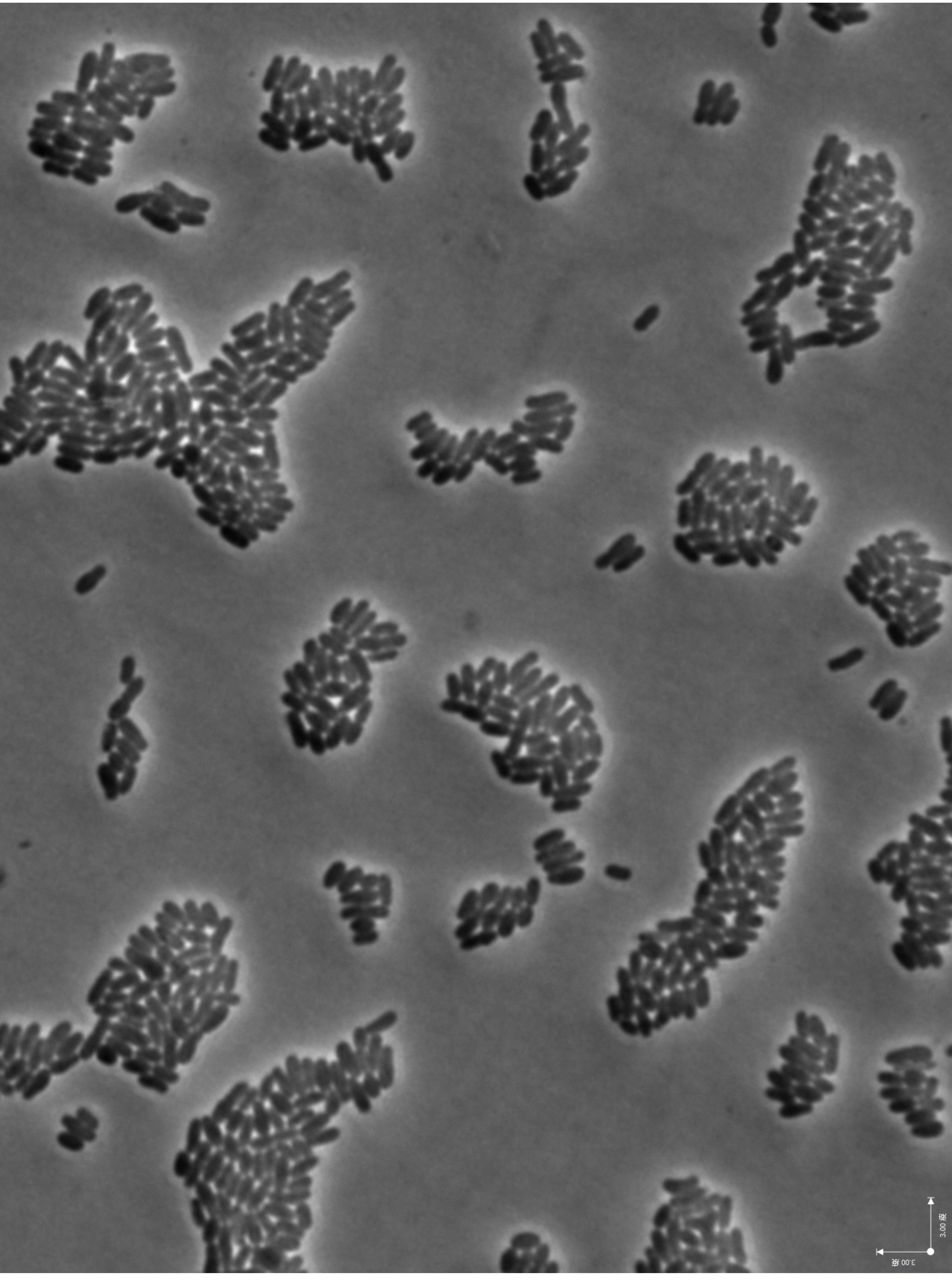

ΔtssLΔtssB (TssB-GFP), GFP

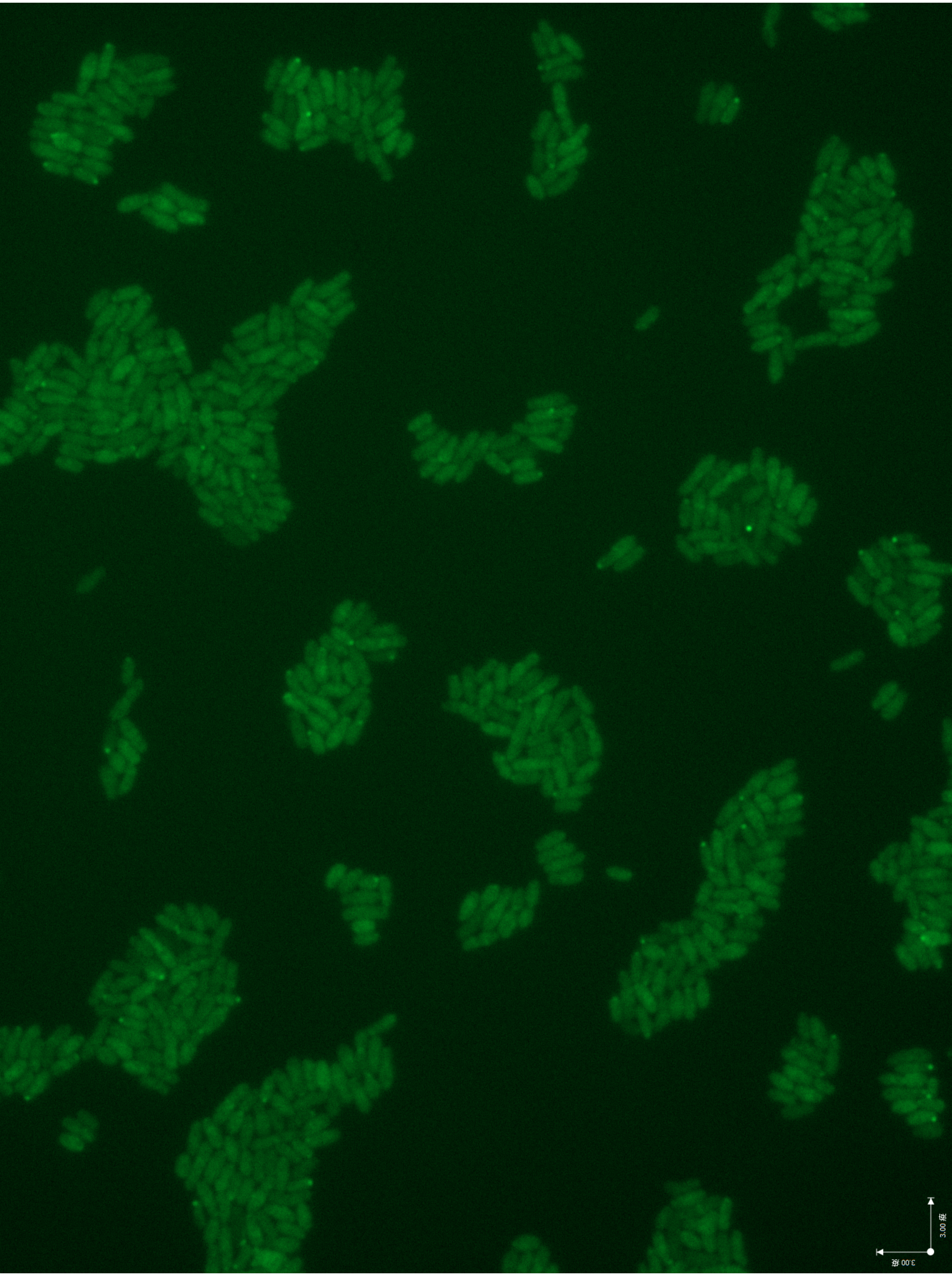

ΔtdeiΔtssB (TssB-GFP), phase contrast

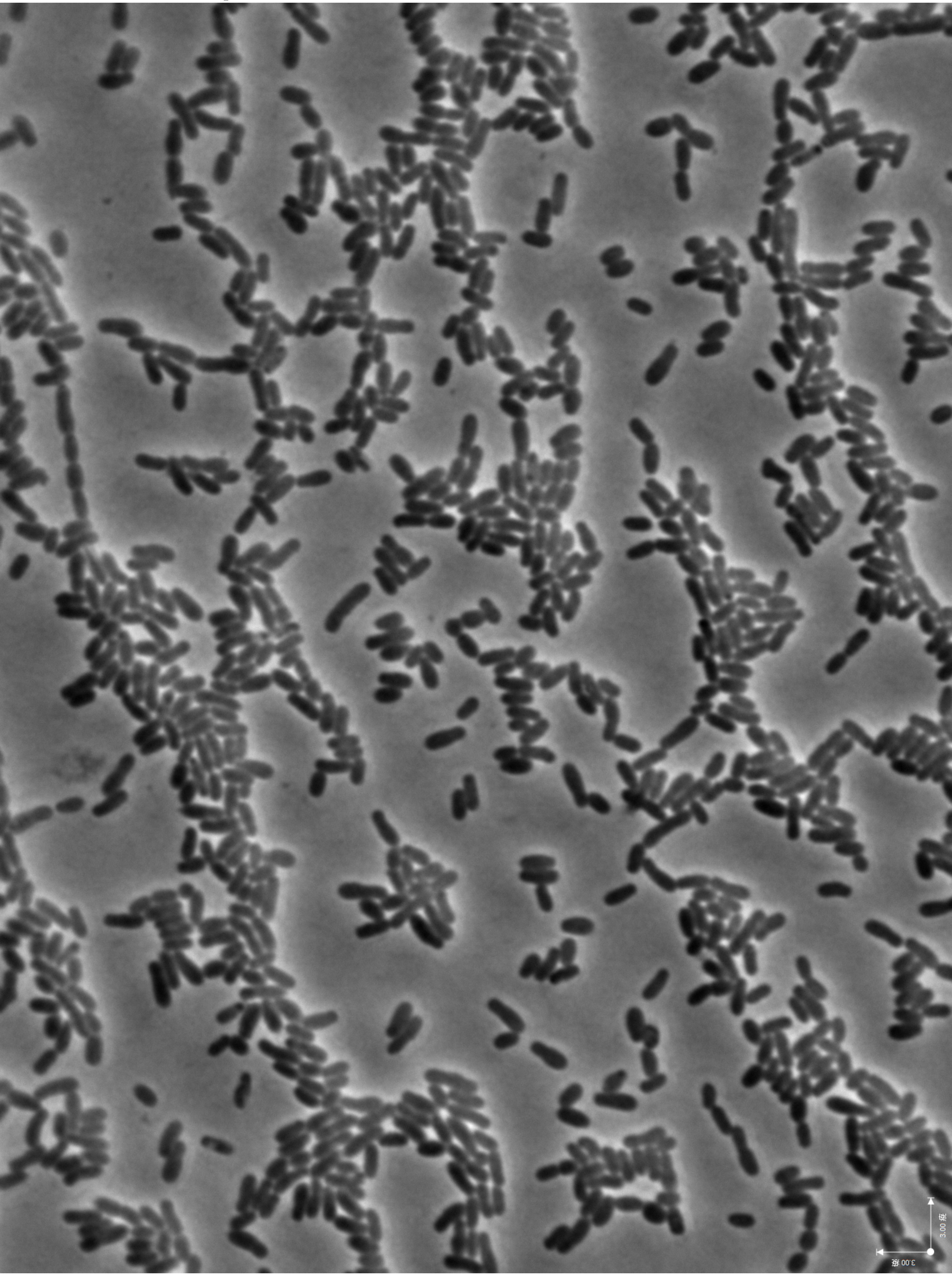

ΔtdeiΔtssB (TssB-GFP), GFP

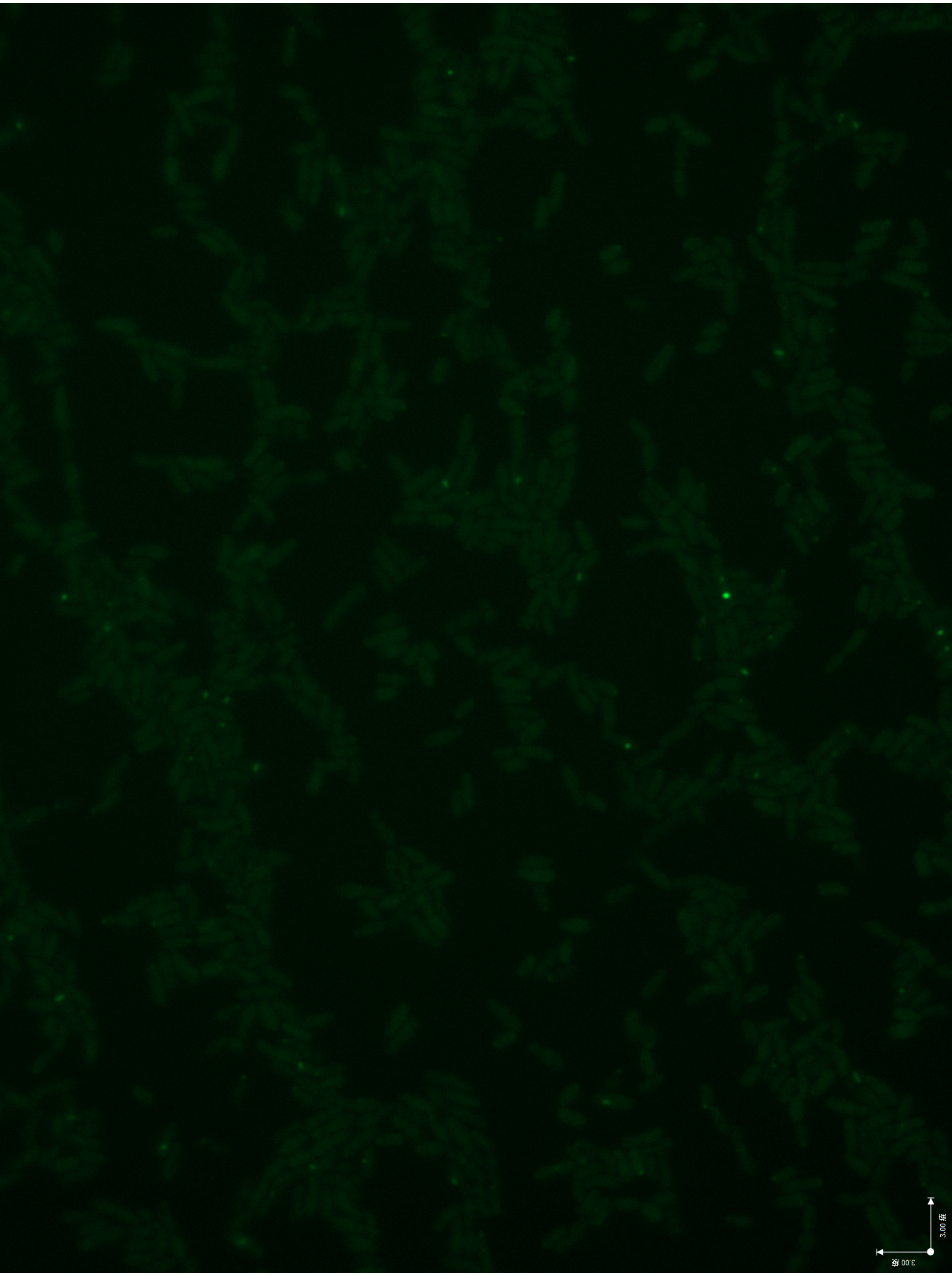

Fig. 3C source data

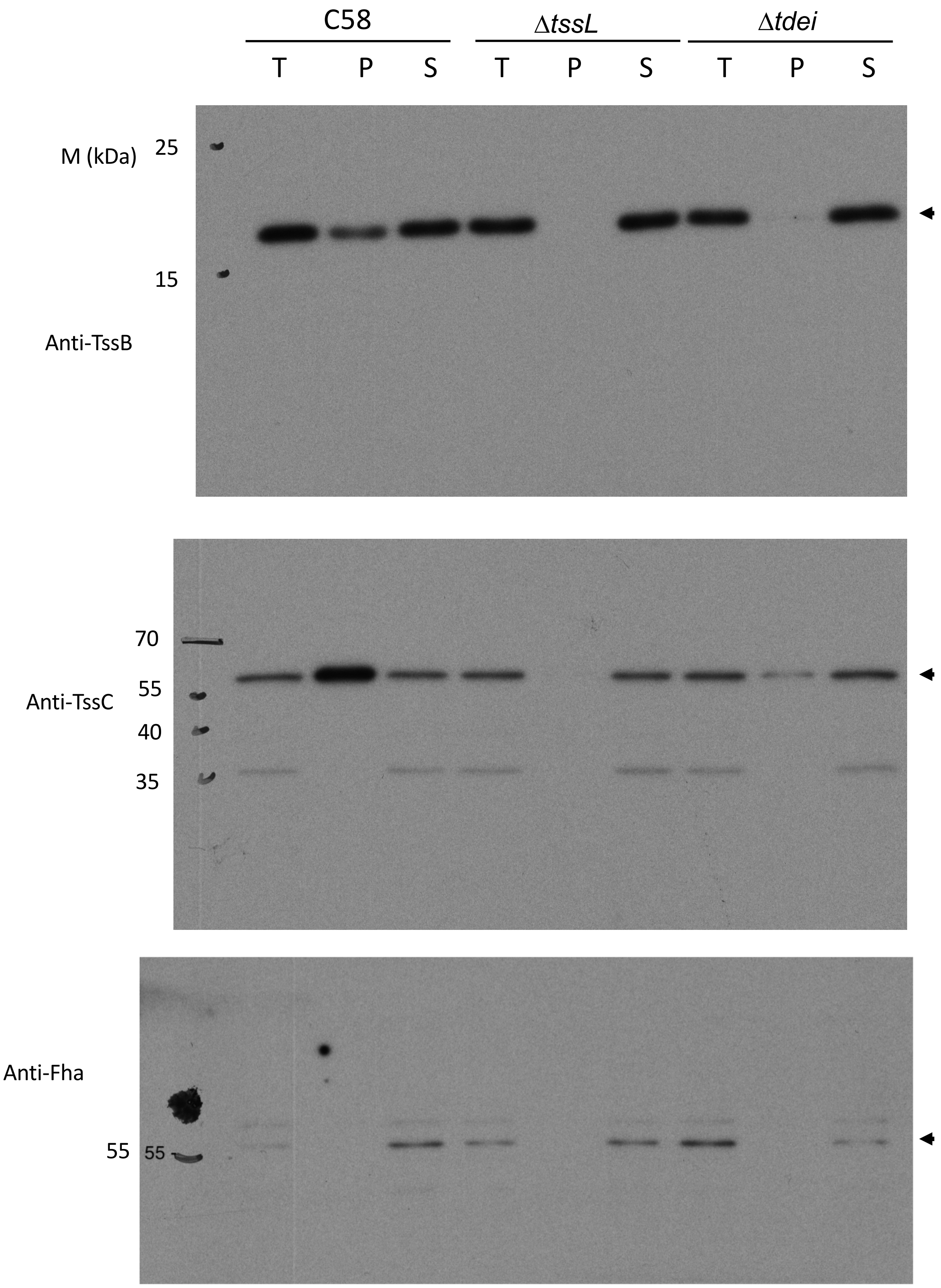

Supplement: Supplementary file 7 — Source Data for Figure 3 [file EMBR-21-e47961-s005.pdf]
